# Remodeling Tumor‐Associated Neutrophils to Enhance Dendritic Cell‐Based HCC Neoantigen Nano‐Vaccine Efficiency

**DOI:** 10.1002/advs.202105631

**Published:** 2022-02-10

**Authors:** Yunhao Wang, Qingfu Zhao, Binyu Zhao, Youshi Zheng, Qiuyu Zhuang, Naishun Liao, Peiyuan Wang, Zhixiong Cai, Da Zhang, Yongyi Zeng, Xiaolong Liu

**Affiliations:** ^1^ The United Innovation of Mengchao Hepatobiliary Technology Key Laboratory of Fujian Province Mengchao Hepatobiliary Hospital of Fujian Medical University Fuzhou 350025 P. R. China; ^2^ Mengchao Med‐X Center Fuzhou University Fuzhou 350116 P. R. China; ^3^ CAS Key Laboratory of Design and Assembly of Functional Nanostructures Fujian Institute of Research on the Structure of Matter Chinese Academy of Sciences Fuzhou 350002 P. R. China; ^4^ Liver Disease Center The First Affiliated Hospital of Fujian Medical University Fuzhou 350005 P. R. China

**Keywords:** dendritic cell‐based vaccine, immunotherapy, neoantigen, neutrophils, photodynamic therapy

## Abstract

Hepatocellular carcinoma (HCC) commonly emerges in an immunologically “cold” state, thereafter protects it away from cytolytic attack by tumor‐infiltrating lymphocytes, resulting in poor response to immunotherapy. Herein, an acidic/photo‐sensitive dendritic cell (DCs)‐based neoantigen nano‐vaccine has been explored to convert tumor immune “cold” state into “hot”, and remodel tumor‐associated neutrophils to potentiate anticancer immune response for enhancing immunotherapy efficiency. The nano‐vaccine is constructed by SiPCCl_2_‐hybridized mesoporous silica with coordination of Fe(III)‐captopril, and coating with exfoliated membrane of matured DCs by H22‐specific neoantigen stimulation. The nano‐vaccines actively target H22 tumors and induce immunological cell death to boost tumor‐associated antigen release by the generation of excess *^1^* O*_2_* through photodynamic therapy, which act as in situ tumor vaccination to strengthen antitumor T‐cell response against primary H22 tumor growth. Interestingly, the nano‐vaccines are also home to lymph nodes to directly induce the activation and proliferation of neoantigen‐specific T cells to suppress the primary/distal tumor growth. Moreover, the acidic‐triggered captopril release in tumor microenvironment can polarize the protumoral N2 phenotype neutrophils to antitumor N1 phenotype for improving the immune effects to achieve complete tumor regression (83%) in H22‐bearing mice and prolong the survival time. This work provides an alternative approach for developing novel HCC immunotherapy strategies.

## Graphical abstract

Acidic/photo‐sensitive dendritic cell‐based neoantigen nano‐vaccine is developed to convert tumor immune “cold” into “hot”, and remodel tumor‐associated neutrophils for enhancing HCC immunotherapy. The nano‐vaccines actively targeted tumors to boost tumor‐associated antigen release, and home to lymph nodes to directly activate neoantigen‐specific T cells. The acidic‐triggered captopril release reduced the protumoral N2 neutrophils for synergetic suppression of the primary/distal tumor growth.

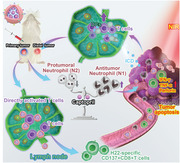

## 1 Introduction

Hepatocellular carcinoma (HCC) ranks the sixth most commonly diagnosed cancer worldwide.^[^ ^1^ ^]^ For HCC patients at the early‐stage, radical resection together with systemic therapy such as targeted therapy is the standard‐of‐care treatment currently; while most HCC patients are commonly diagnosed at the advanced stage, therefore are not suitable for surgical operation, and suffered from very poor prognosis and therapeutic responses to standard treatments.^[^ ^2^, ^3^ ^]^ Recently, with the rapid progress of immunotherapy, immune checkpoint inhibitors (ICIs) (anti‐programmed cell death 1 (PD1) or anti‐PD1 ligand 1 (PDL1) antibodies) have been extensively applied to control the progression of advanced HCC, even with distant metastasis (lung, bones, or brain).^[^ ^4^, ^5^, ^6^ ^]^ However, the overall response rate of HCC patients to ICIs is relatively low (less than 20%) and only a small portion of HCC patients could benefit from ICI treatment, most likely due to the “cold” state of HCC tumors with relatively low immunogenicity, restricted tumor‐infiltrating lymphocytes (TILs), and inhibitory immune microenvironment.^[^ ^7^, ^8^ ^]^ Therefore, it is highly desired to develop novel immunotherapy strategies to systematically switch the “cold” nature of HCC tumor into “hot”, and re‐shape the unfavorable immune micro‐environment for improving the therapeutic response rate and long‐term outcomes.^[^ ^9^ ^]^

Therapeutic cancer vaccine (TCVs), as a promising and alternative cancer immunotherapeutic strategy to induce tumor regression, eradicate minimal residual disease, establish lasting antitumor memory and avoid strong adverse effects, has achieved remarkable success over the past decade through exploiting the patients’ immune system to fight against tumor.^[^ ^10^, ^11^, ^12^ ^]^ Recently, neoantigens that expressed specific genome somatic mutations of tumors have attracted much attention as the famous targets for developing TCVs, due to its extremely low off‐target effects, high specificity, and strong immunogenicity without pre‐existing central tolerance.^[^ ^13^, ^14^, ^15^ ^]^ Accordingly, neoantigen‐based dendritic cell (DC) vaccine (neo‐DCs) has shown great potential, since it could directly induce strong immunogenicity to activate T cell immune response to kill cancer cells and suppress tumor progression with low toxicity, which has been verified in the various pre‐clinic/clinical trials worldwide.^[^ ^16^, ^17^ ^]^ Whereas the overall clinical responses of neo‐DCs are still unsatisfactory and need to combine with other treatment modalities to further improve its potency.^[^ ^18^, ^19^, ^20^ ^]^ Interestingly, recent studies showed that peritumoral infiltration of neutrophils has positively correlated with the angiogenesis progression at the tumor‐invading edge of HCC patients, and the angiotensin‐converting enzyme inhibitor can repress tumor growth through polarization of pro‐tumorigenic N2 phenotype neutrophils to an antitumoral phenotype (N1 phenotype).^[^ ^21^, ^22^, ^23^ ^]^ Therefore, combination of neo‐DCs with TAN remodeling may provide an attractive approach to potentiate the therapeutic outcomes of neoantigen vaccines.

As vaccine or drug delivery vectors, the prevalent biomimetic nanosystems that functionalized with immune cell membrane have held great strengths for disease‐targeted therapy with minimized side effects, such as unique tumor tropism, prolonging the circulation, recognizing specific targets, enhancing intercellular interaction, and low system toxicity;^[^ ^24^, ^25^, ^26^, ^27^, ^28^, ^29^, ^30^ ^]^ meanwhile, they also could easily integrate with other therapeutic modules for synergistic treatment of cancers.^[^ ^31^, ^32^ ^]^ Among the commonly used therapeutic modules, local photodynamic therapy (PDT) can enhance the immunogenic cell death (ICD) of target tumors, characterized by the release of damage‐associated molecular patterns (DAMPs) through near‐infrared (NIR) laser mediated *^1^* O*_2_* oxidation; these released ICD molecules can further act as in situ vaccination to elicit systemic antitumor immunity at a certain degree to overcome low immunogenicity of “cold” tumors.^[^ ^33^, ^34^ ^]^ Nevertheless, most of the traditional photosensitizing agents for PDT always suffered from weak hydrophilicity, low photostability, and insufficient tumor accumulation, which lead to unsatisfactory *^1^* O*_2_* generation and limited killing efficiency to tumors with insufficient ICD.^[^ ^35^ ^]^ Recently, we have developed a one‐step method to enhance the water solubility and photo‐stability of black phosphorus quantum‐dot photosensitizer through mesoporous silica framework (MSF) as a matrix,^[^ ^36^ ^]^ and it subsequently showed relative high accumulation into tumors after coating with lymphocyte membranes,^[^ ^26^ ^]^ which might provide an effective idea for improving the ICD of PDT treatment.

**Figure 1:**
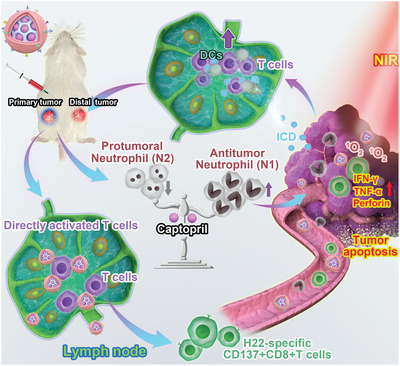
Schematic illustration of remodeling tumor‐associated neutrophils to enhance DC‐based HCC neoantigen nano‐vaccine efficiency. The H22 liver cancer cell‐specific neoantigens are predicted by in silico analysis and confirmed through ELISPOT. Afterward, the neoantigen activated DC‐based nano‐vaccines are prepared, which can not only actively target H22 tumor tissues to enhance TAA release through PDT but achieved the lymph‐homing ability to directly induce the activation and proliferation of CD8+T cells. These led to strengthening the immune responses against the primary and distant tumor growth. More strikingly, the tumor acidic‐triggered release of captopril can reduce the protumoral N2 phenotype to further improve the immune effects to further augment the suppression of both the primary and distance tumor growth, therefore prolonging the survival of H22‐bearing mice.

In the present work, the silicon phthalocyanine dichloride (SiPCCl_2_) embedded mesoporous silica, (named SMN), was reported to act as the nano‐photosensitizing agent; Fe(III)‐captopril complexes loaded into the SMN through the inherent porous nanostructure and coordination effect, served as the acidic‐responsive nanodrugs for TAN modulation (**Figure** **1**). After coating with exfoliated membrane of matured DCs by H22 mouse liver cancer‐specific neoantigen stimulation, the nano‐ vaccine was obtained (named mD@cSMN) with the prominent tumor‐targeting ability and lymph‐homing effect. In the H22 tumor‐bearing mice, the effective accumulation of nano‐vaccines at the tumor site could enhance the ICD effect through in situ generation of *^1^* O*_2_* upon the NIR laser irradiation for eliciting TAA‐specific T cell immune responses; simultaneously, the lymph‐homing of nano‐vaccines could also directly induce the tumor‐specific antigen (TSA)‐specific T cell activation and proliferation to against tumor. Notably, the tumor acidic‐triggered captopril release from mD@cSMN could further polarize the protumoral N2 phenotype neutrophils to antitumor N1 phenotype to reverse the inhibitory immune micro‐environment in tumors, thereafter synergistically suppressing both the primary and distal tumor proliferation to prolong the survival of H22 mouse liver cancer‐bearing mice. This work may offer valuable insights to developing TCVs for potentiating HCC immunotherapy in future.

## 2 Results and Discussion

### 2.1 Synthesis and Characterization of mD@cSMNs

**Figure 2:**
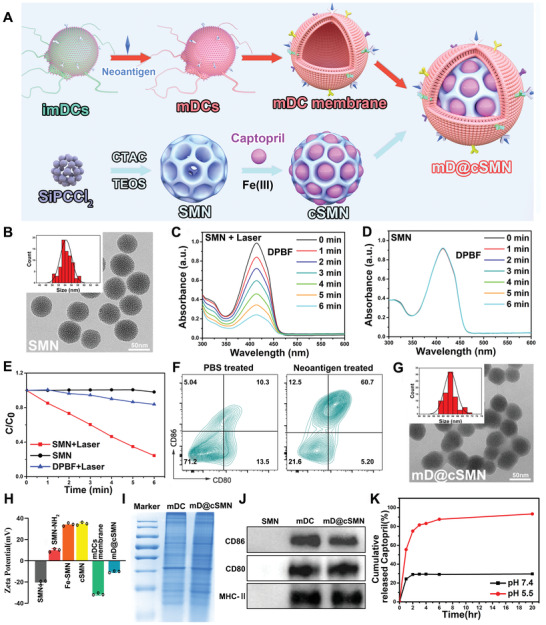
Characterization of mD@cSMN nano‐vaccines. A) Schematic illustration of the preparation of mD@cSMN nano‐vaccines. B) TEM image of SMN photosensitizers and the size distribution of SMNs (insert picture). C) The absorbance of DPBF after decomposition by generated *^1^* O*_2_* from SMN with and without D) 670 nm laser irradiation (50 mW cm^−2^) for different times. E) The normalized absorbance of DPBF at 415 nm after decomposition by ROS generation in SMNs with or without irradiation for different times and the DPBF without SMN is used as the control. F) The maturation of BMDCs after co‐incubation with PBS or H22 tumor cell‐specific neoantigen for 72 h, respectively, which are analyzed by FACS with staining CD80 and CD86 antibodies. G) The TEM image of mD@cSMN nano‐vaccines and their size distribution (insert picture). H) The surface zeta potential of the SMNs, SMNs‐NH_2_, Fe‐SMNs, cSMNs, the mature DCs membrane, and mD@cSMNs, (*n* = 3). I) The protein pattern analysis of matured DCs membrane and mD@cSMNs through SDS‐PAGE (coomassie blue staining). J) Western blotting analysis of membrane‐specific protein markers. The samples are run at equal protein amounts and blotted with CD80, CD86, and MHC‐II antibodies. K) The cumulative captopril release kinetics from mD@cSMNs in different pH conditions within 20 h.

Considering the capacity of PDT to promote the release of DAMPs for APC activation, we herein designed a water‐stable nano‐agent as photosensitizer (SMN), which was constructed by SiPCCl_2_ (*Φ*_Δ_ = 0.15, commonly used as PDT agent^[^ ^37^ ^]^) embedded into the matrix of MSF through Si—O—Si bond between SiPCCl_2_ and tetraethyl orthosilicate (TEOS) to endow the SiPCCl_2_ with water‐stability and prevent its leakage during in vivo circulation (**Figure** **2**A). Compared with the SiPCCl_2_ dissolved in PBS buffer with a low and broad absorbance (600 to 900 nm) attributing to the aggregation of hydrophobic SiPCCl_2_, our prepared SMNs showed a typical absorbance from 650 to 700 nm in PBS solution as similar as SMNs dissolved in EtOH, and it was also in accordance to the SiPCCl_2_ dissolved in EtOH (Figures S1 and S2, Supporting Information). The transmission electron microscopy (TEM) image of SMNs showed a spherical morphology with an average diameter of 54.30 ± 6.81 nm (Figure 2B and Figure S3, Supporting Information). Barrett–Joyner–Haenda (BJH) desorption showed the average pore diameter of 10.19 nm of SMNs and the recorded BET surface area about 651.7116 m^2^ g^−1^ through the BJH model, suggesting the loading capacity of SMNs (Figures S4 and S5, Supporting Information).

Next, the capacity of singlet oxygen (*^1^* O*_2_*) production of SMNs nanophotosensitizers was first investigated by 1,3‐diphenylisobenzofuran (DPBF) probe as the *^1^* O*_2_* detector.^[^ ^38^ ^]^ After irradiated by 670 nm with the power intensity of 50 mW cm^−2^, the absorbance of DPBF probe in the presence of SMN was significantly decreased with a time‐dependent behavior in comparison to DPBF probe alone with laser or DPBF probe in the presence of SMN without NIR laser irradiation, suggesting the significant *^1^* O*_2_* generation ability of the prepared SMNs (Figure 2C–E and Figure S6, Supporting Information). Furthermore, to evaluate the photo‐stability of SMNs, the SMN solution was repeatedly irradiated by NIR laser (50 mW cm^−2^) with ON/OFF cycles (irradiated for 2 min in 1 min intervals) for 8 times, and analyzed by UV–vis–NIR absorption spectra. As shown in Figure S7, Supporting Information, the absorption of SMNs after repeated laser irradiation still kept their stability without any change, indicating an excellent photo‐stability of our prepared SMN nanophotosensitizers. In contrast, the absorption of SiPCCl_2_ in EtOH was sharply decreased after repeated irradiation at the same conditions (Figure S8, Supporting Information). The results demonstrated that SMNs with excellent water‐solubility, photostability, and ROS generation ability could act as a new nanophotosensitizer for PDT.

Tumor microenvironment (TME) sensitive drug delivery nanosystems have provided a promising strategy for “on‐demand” release of conventional drugs to avoid side effects.^[^ ^39^ ^]^ Here, we introduced host‐metal‐guest complexes to efficiently load the captoprils into SMNs (cSMN) by Fe(III)‐mediated coordination effects according to our previously reported method.^[^ ^40^ ^]^ TEM image of cSMN still showed a typical spherical morphology with good dispersion (Figure S9, Supporting Information). Besides, the existence of Fe element in cSMNs was proved through HAADF‐STEM, and the content of captopril was quantified by HPLC (Figures S10 and S11, Supporting Information). Finally, the H22 liver cancer cell specific neoantigen (identified by our recently reported work^[^ ^41^ ^]^) was used to stimulate the immature bone marrow‐derived DCs, then the matured DCs (mDCs) were verified through FACS with staining CD80 and CD86 antibodies (Figure 2F). Then, the mDCs membrane with overexpression of major co‐stimulatory molecules (CD80 and CD86) were exfoliated and coated on the surface of cSMNs to obtain mD@cSMNs. The obtained mD@cSMNs were visualized by TEM image which showed an average diameter of 63.01 ± 2.61 nm with a membrane thickness of ≈8.7 nm (ImageJ software analysis) (Figure 2G and Figure S12, Supporting Information). To verify the successful coating of mDCs membrane on the cSMNs, we initially measured the zeta potential of mD@cSMNs. As shown in Figure 2H, the potential of mD@cSMNs was significantly changed from +36.30 mV (cSMN) to −9.71 mV likely due to the negative change of purified mDC membrane (−31.47 mV). Furthermore, the specific bio‐markers of mDC membrane were also investigated by SDS‐PAGE (coomassie brilliant blue staining) and western blot, respectively. The results showed that our mD@cSMNs displayed the enrichment of CD80, CD86, and major histocompatibility complex (MHC‐II) markers which are similar to the purified mDC membrane, indicating the successful coating of mDC membrane on the mD@cSMNs (Figure 2I,J and Figure S13, Supporting Information). Besides, we further studied the water stability of SMNs by DLS. The results showed that the hydrodynamic size and polydispersion index of SMNs did not significantly change during the co‐incubation periods for 5 days at room temperature, indicating the excellent water‐stability of mD@cSMNs (Figure S14, Supporting Information). Next, the TME acidic‐triggered captopril release profile of mD@cSMNs was investigated and quantified by HPLC under different mimic pHs (7.4 and 5.5). The released percentage of captoprils from mD@cSMNs was only 28.91% at pH 7.4 after 20 h incubation (Figure 2K); however, the released percentage of captoprils in mD@cSMNs at pH 5.5 was remarkably increasing up to 91.02% after 20 h incubation, indicating the high sensitivity to TME acidic condition of our mD@cSMNs for “on‐demand” drug release.

### 2.2 Photodynamic Enhancement of ICD Effect and Induction of DC Maturation In Vitro

**Figure 3:**
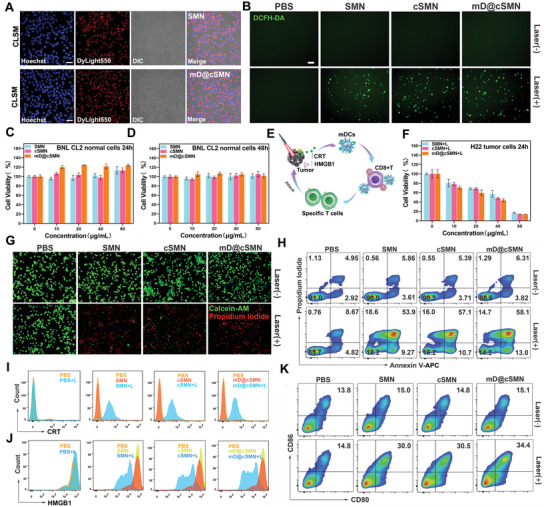
Photodynamic enhancement of ICD and activation of immune responses in vitro. A) CLSM images of H22 liver cancer cells uptaking Dylight 550‐NHS labeled SMNs or mD@cSMN nano‐vaccines. The blue is represented Hoechst33342, and the red is represented Dylight550. Scar bar, 100 µm. B) CLSM images of intracellular ROS generation of PBS, SMNs, cSMNs, and mD@cSMNs with or without NIR laser irradiation (50 mW cm^−2^) for 5 min, respectively. DCFH‐DA acted as a ROS fluorescence indicator (green, excited by 488 nm). Scar bar, 100 µm. C,D) The Cell viability of BNL CL2 normal liver cells treated with various doses of PBS, SMNs, cSMNs, and mD@cSMNs after 24 or 48 h coincubation, in dark conditions, respectively, (*n* = 5). E) Schematic illustration of antitumor effect through mD@cSMNs upon the 670 nm laser irradiation and maturation of BMDCs through detecting CRT and high‐mobility group box 1 protein (HMGB1) in vitro. F) The Cell viability of H22 cells treated with different doses of PBS, SMNs, cSMNs, and mD@cSMNs with NIR laser irradiation for 5 min, respectively. The Cell viability is analyzed by CCK8 kit, (*n* = 5). G) Fluorescence images of the live/dead cell viability assay kit (Calcein‐AM/PI) stained H22 cells with or without laser irradiation as indicated treatments. Scar bar, 100 µm. H) The apoptosis/necrosis of H22 cells is analyzed by FACS with staining Annexin‐V‐APC/PI under the following conditions: PBS treated H22 cells with or without 670 nm laser irradiation (50 mW cm^−2^) for 5 min; H22 cells incubated with SMNs with or without laser irradiation; H22 cells incubated with cSMNs with or without laser irradiation; H22 cells incubated with mD@cSMNs with or without laser irradiation; The surface‐exposed CRT (I) and released HMGB1 (J) detection from PBS, SMN, cSMN, or mD@cSMN treated H22 cancer cells with or without laser irradiation, which are analyzed by FACS. K) The maturation of BMDCs after co‐incubation with PBS, SMN, cSMN, or mD@cSMN treated H22 cancer cells for 24 h, respectively. Data are presented as mean ± SEM.

Limited tumor‐associated antigen (TAA) release of “cold” tumors is significantly hindered the host immune responses against tumors after receiving primary therapies. To overcome this issue, PDT has been recognized as an efficient approach to improve both the innate and adaptive immunity basing on the enhancement of the ICD effect.^[^ ^34^ ^]^ We next investigated the ICD enhancing capacity of PDT by our prepared mD@cSMNs under the NIR laser irradiation. Firstly, the bio‐safety of mD@cSMNs was evaluated by hemolysis assay. As shown in Figures S15 and S16, Supporting Information, we found that no major impact on hemolysis in mD@cSMN or SMN treated mouse red blood cells, indicating a good hemocompatibility of mD@cSMNs. After co‐incubated with H22 cells for 24 h, confocal microscopy (CLSM) images showed the strong red fluorescence in Dylight550 labeled mD@cSMNs treated H22 cells, and these findings were similar to that of Dylight550 labeled SMNs treated H22 cells (**Figure** **3**A and Figure S17, Supporting Information). It suggested the efficient cellular uptake of mD@cSMNs nano‐vaccines by H22 cancer cells, which might benefit the intracellular ROS generation in H22 cancer cells upon laser irradiation. To confirm this hypothesis, the intracellular ROS generation from nano‐vaccine treated H22 cancer cells or tumors under the NIR laser irradiation (50 mW cm^−2^) was investigated, respectively. 2, 7‐dichlorodihydroflfluorescein diacetate (DCFH‐DA) was taken as the ROS fluorescence indicator. The PBS‐treated H22 cancer cells with NIR laser irradiation were used as the control. As shown in Figure 3B and Figure S18, Supporting Information, SMN, cSMN, or mD@cSMNs treated H22 cells with NIR laser irradiation all showed significant ROS production in H22 cancer cells with the obvious green fluorescence comparing to that of the PBS‐treated cells at the same conditions, and similar results were also obtained in the freezing *ex* tumor tissue slices from mD@cSMNs treated groups with NIR laser irradiation for 5 min, indicating that mD@cSMNs could act as a new photodynamic agent for PDT.

To further investigate the toxicity of mD@cSMNs in normal liver cells (BNL CL2) in dark conditions to prove the bio‐safety of our mD@cSMN nano‐vaccines, CCK8 kit was conducted. As shown in Figure 3C,D, a different formulation including the SMNs, cSMNs, or mD@cSMNs treated BNL CL2 cells with different concentrations did not impact BNL CL2 cell viability with the viable cells almost 100% in dark conditions, and the results were further confirmed after prolonging the co‐incubation time up to 48 h. Whereas, upon NIR laser irradiation (50 mW cm^−2^) for 5 min, the cell viability of H22 cells was decreasing sharply from 100% to 13.79% in 80 µg mL^−1^ of mD@cSMNs treated cells, and the results were similar to the SMN (17.25%) or cSMN (14.08%) treated groups, suggesting the direct killing efficiency in vitro of mD@cSMNs mainly resulting from the generated *^1^* O*_2_* from the SMN core (Figure 3E,F). These findings were further proved by the localized photo‐killing experiments under NIR irradiation and then analyzed by the live/dead viability/cytotoxicity kit staining (Calcein‐AM/PI) (Figure 3G). To calculate the apoptosis or necrosis rate, the H22 cells were treated with SMNs, cSMNs, or mD@cSMNs, and subsequently staining by AnnexinV‐APC/PI and analyzed by flow cytometry. As shown in Figure 3H, 90.46% and 85.33% of viable cells were observed in PBS treated H22 cells with or without NIR laser irradiation; In contrast, only 10.74% of viable cells was detected in mD@cSMN treated H22 cells after NIR irradiation for 5 min, and the results were similar with the SMN or cSMN treated H22 cells at the same conditions. These findings demonstrated that our prepared mD@cSMNs could act as a bio‐safe photosensitizer for efficiently killing H22 cancer cells without other unfavorable effects.

Tumor cell surface‐exposed calreticulin (CRT), actively secreted adenosine triphosphate (ATP), and released high‐mobility group box 1 protein (HMGB1) which have been considered as the biomarkers of ICD were further investigated.^[^ ^42^ ^]^ After staining the CRT antibody, different formulations including the SMNs, cSMNs, or mD@cSMNs all enabled to effectively induce the generation of tumor cell surface‐exposed CRT, comparing with the PBS‐treated groups after NIR laser irradiation (Figure 3I). Similarly, other ICD markers such as the amount of released HMGB1 and secreted ATP from the SMN, cSMN or mD@cSMN treated H22 cells were also significantly increased, clearly demonstrating the enhancement of ICD through PDT (Figure 3J and Figure S19, Supporting Information). Afterward, the DAMPs released from mD@cSMNs treated H22 cancer cells (in the supernatant) were then co‐incubated with immature BMDCs for 24 h, and analyzed by FACS with staining the CD80 and CD86 antibodies. As shown in Figure 3K and Figures S20 and S21, Supporting Information, compared to the PBS‐treated groups (including both with or without laser irradiation), the mD@cSMNs treated group with laser irradiation could efficiently enhance the maturation of DCs (34.4%) indicating by the increase of CD80+CD86+ proportion cells. These results demonstrated the capacity of mD@cSMNs to enhance ICD effect of cancer cells by PDT for effectively activating the maturation of DCs.

### 2.3 Nanovaccine Induced T cell Activation and Proliferation for Effectively Killing H22 Cells In Vitro

**Figure 4:**
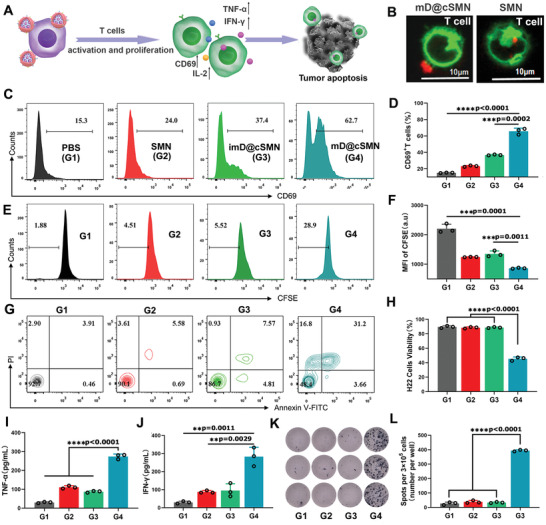
Directly activation of T cell proliferation and effective killing of H22 cells in vitro. A) Schematic illustration and B) CLSM image of the interaction between mD@cSMNs and inactivated T cells from BALb/c mice spleen, which directly induced the T cell activation and proliferation as well as cytokine secretion. Green fluorescence is represented Dil labeled T cells and red fluorescence is represented Dylight550‐NHS labeled mD@cSMNs. C,D) The activation of T cells after co‐incubation with PBS, SMNs, imD@cSMNs, or mD@cSMNs for 72 h, respectively, and analyzed by FACS with staining CD69 and CD3, (*n* = 3). E,F) The proliferation of CSFE labeled CD8+T cells after co‐incubation with PBS, SMNs, imD@cSMNs, or mD@ cSMNs, respectively, and analyzed by FACS, (*n* = 3). G,H) The toxicity of T cells that activated by PBS, SMNs, imD@cSMNs, or mD@cSMNs after co‐incubation with H22 cells for 72 h, and analyzed by FACS with staining Annexin V‐FITC and PI, (*n* = 3). The secretion of TNF‐*α* (I) and IFN‐*γ* (J) is checked by ELISA kit after 72 h of post‐incubation with H22 cells, (*n* = 3). K,L) ELISPOT analysis of IFN‐*γ* spot‐forming of PBMCs cells from BALb/c after s.c. injection of PBS, SMNs, imD@cSMNs, or mD@cSMNs for 14 days, and then co‐incubated for 48 h (*n* = 3). G1, PBS; G2, SMN; G3, imD@cSMN; G4, mD@cSMN. The statistical analysis is performed with ANOVA analysis, *p < 0.05, **p < 0.01, ***p < 0.001, ****p < 0.0001, (*n* = 3). Data are presented as mean ± SEM.

Noteworthy, the neo‐DCs have been used to act as an effective antitumor modality to stimulate patients’ immune response through utilizing TSA and specifically kill the tumor cells carrying neo‐mutations.^[^ ^16^ ^]^ We, thus, assessed the impact of mD@cSMN nano‐vaccines on the activation of T cells. An immature DC membrane coating on the surface of cSMNS (refer to imD@cSMNS) that co‐incubated with inactivated T cells from BALB/c mouse spleen was used as the control. As shown in **Figure** **4**A,B, based on antigen capture, the mD@cSMNs with red fluorescence directly interacted with the inactivated T cells with green fluorescence instead of devouring the non‐immunogenic SMNs, might be ready to elicit T cell responses. Thereafter, the FACS studies showed that mD@cSMN nano‐vaccine treated T cells could induce a significantly higher population of co‐stimulatory receptor overexpression (CD3+CD69+) in T cells than other treatments, indicating the capacity of enhanced immune stimulation and T cell activation mediated by mD@cSMNs (Figure 4C,D). Moreover, the mD@cSMN nano‐vaccines could also effectively promote T cell proliferation through analyzing the CFSE‐labeled T cells (Figure 4E,F). Given the above results of mD@cSMN nano‐vaccines on activating CD69+T cells, the killing efficiency of these activated T cells mediated by mD@cSMN nano‐vaccines was then investigated through co‐incubating with H22 cancer cells. The cell apoptosis or necrosis of H22 cells after co‐incubation was quantified by FACS with staining Annexin V‐FITC/PI. As shown in Figure 4G,H, and Figure S22, Supporting Information, the apoptosis and necrosis percentage of tumor cells were increased up to 51.6% after 36 h co‐incubation with mD@cSMN treated T cells, which were higher than that of imD@cSMN (13.3%) nano‐vaccine treated T cell group. Moreover, the inflammatory cytokines secreted by activated T cells after incubating with H22 tumor cells were detected by EILSA. As shown in Figure 4I,J and Figure S23, Supporting Information, the significantly higher levels of TNF‐*α*, IFN‐*γ*, and IL‐2 in co‐incubation group of mD@cSMN treated T cells with H22 cells were observed compared to the cSMN or imD@cSMN treated groups. Furthermore, to verify the immunological stimulation of mD@cSMN nano‐vaccine in vivo, an ELISpot assay was performed through s.c. injection of nano‐vaccine into BALB/c mice for 14 days. As shown in Figure 4K,L, mD@cSMN nano‐vaccines could more efficiently promote the secretion of IFN‐*γ* than that of other treatments, indicating the excellent T cell activation for responding to mD@cSMN nano‐vaccine. These findings demonstrated that our mD@cSMN nano‐vaccines enabled to direct induction of T cell activation and proliferation to efficiently kill the H22 liver cancer cells.

### 2.4 Remodeling Tumor‐Associated Neutrophils to Enhance Immunotherapy Efficiency

**Figure 5:**
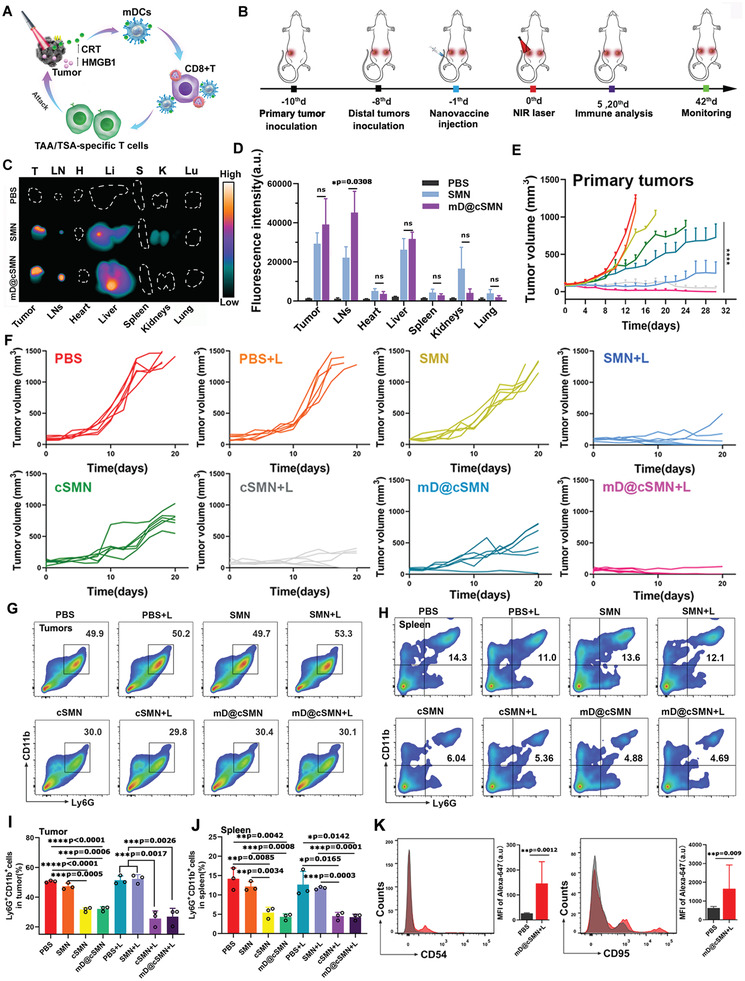
Active targeting H22 tumors and LNs homing effect to effectively inhibit tumor growth through mD@cSMN nano‐vaccines by activating immune responses under the 670 nm laser irradiation. A,B) Schematic illustration of antitumor effect by mD@cSMN nano‐vaccines and their administration procedure in the established primary H22 tumor‐bearing mice and distal tumors upon the laser irradiation. C) Ex vivo fluorescence images of tumors, LNs, and major organs that are isolated from H22 tumor‐bearing mice after 24 h of ICG‐labeled SMNs and mD@cSMN injection, and the PBS treated mice is used as the control. D) Fluorescence intensity of tumors, LNs, and major organs after s.c. injection of nano‐vaccines at 24 h, (*n* = 3). The statistical analysis is performed with two‐tail paired Student‘s *t*‐test analysis, *p < 0.05. ns means *p >* 0.05. E,F) The primary tumor volume change of mice after PBS, SMN, cSMN, or mD@cSMN treated mice with or without 670 nm irradiation (0.1 W cm^−2^) of primary tumors for 5 min (*n* = 6). The percentage of CD11b+Ly6G+TANs in the primary tumors (G,I) and spleen (H,J) are investigated after receiving different treatments as indicated on the 20th day (*n* = 3). K) Cell surface expression of typical N1 neutrophil markers (CD54 and CD95) in tumors after treated with mD@cSMN+NIR for 20 days, and then analyzed by flow cytometry with staining CD54 and CD95 antibodies, (*n* = 3). The statistical analysis is performed with ANOVA analysis, *p < 0.05, **p < 0.01, ***p < 0.001, ****p < 0.0001. Data are presented as mean ± SEM.

Next, the synergistically therapeutic efficiency of mD@cSMN nano‐vaccines including PDT, anti‐cancer immune response, and captopril in vivo, were carefully investigated (**Figure** **5**A,B). Firstly, to enhance the PDT efficiency, the excellent actively targeting capacity to tumors of nanodrug was essential.^[^ ^36^ ^]^ After subcutaneous inoculation of ICG‐NHS labeled mD@cSMN nano‐vaccines for 24 h, the tumor, LNs, heart, liver, spleen kidney, and lung were isolated and then imaged. As shown in Figure 5C,D, the relative high fluorescence intensity of ICG‐NHS labeled mD@cSMNs was observed in H22 tumors, compared with the subcutaneous (s.c) inoculation of ICG‐NHS labeled SMNs without coating the mDC membrane, suggesting an efficient tumor‐targeting ability of mD@cSMN nano‐vaccines, which would benefit for improving the PDT therapeutic efficiency. Noteworthy, compared with the bio‐distribution of ICG‐NHS labeled SMNs in LNs, the mD@cSMN treated mice showed high accumulation in LNs with relative higher fluorescence intensity, suggesting an excellent LNs‐homing effect of mD@cSMNs, which would achieve an efficient systemic anti‐cancer immune response.

To confirm the above assumption, we established a dual‐tumor model by s.c. injecting H22 tumor cells into the left and right groin of BALB/c mice as the models of primary and distal tumors, respectively. The mice model were randomly divided into 8 groups (*n* = 12 per group) including the PBS, PBS + NIR, SMN, SMN + NIR, cSMN, cSMN + NIR, and mD@cSMN, mD@cSMN +NIR, respectively. The 670 nm laser with the power intensity of 0.1 W cm^−2^ for irradiated 5 min was utilized in in vivo experiments after s.c. injection of different formulations for 24 h. The tumor volume was measured by vernier caliper. As shown in Figure 5E,F, as expected, rapid tumor growth in the primary tumors was detected in the PBS, PBS+NIR, and SMN, while the mice inoculated with cSMN alone could delay the primary tumor growth largely due to the reduction of the protumoral N2 phenotype neutrophils in tumors to improve immune effect against tumor cells through tumor acidic‐triggered release of captoprils from cSMN.^[^ ^22^ ^]^ Meanwhile, the primary tumors were also suppressed in the SMN + NIR group compared to the PBS + NIR group attributing to the PDT effect. In addition, an efficient suppression of primary tumor growth was observed in mD@cSMNs alone compared to other groups without NIR irradiation mainly attributing to the captopril functions and direct activation of antitumor T cell immunity (ELISpot assay in vivo). More obviously, the antitumor efficiency of the primary tumor in mD@cSMNs + NIR group was significantly higher than that of SMN +NIR group or cSMN+NIR group mainly attributing to the synergistic functions of PDT, captopril, and direct activation of antitumor T cell immunity.

**Figure 6:**
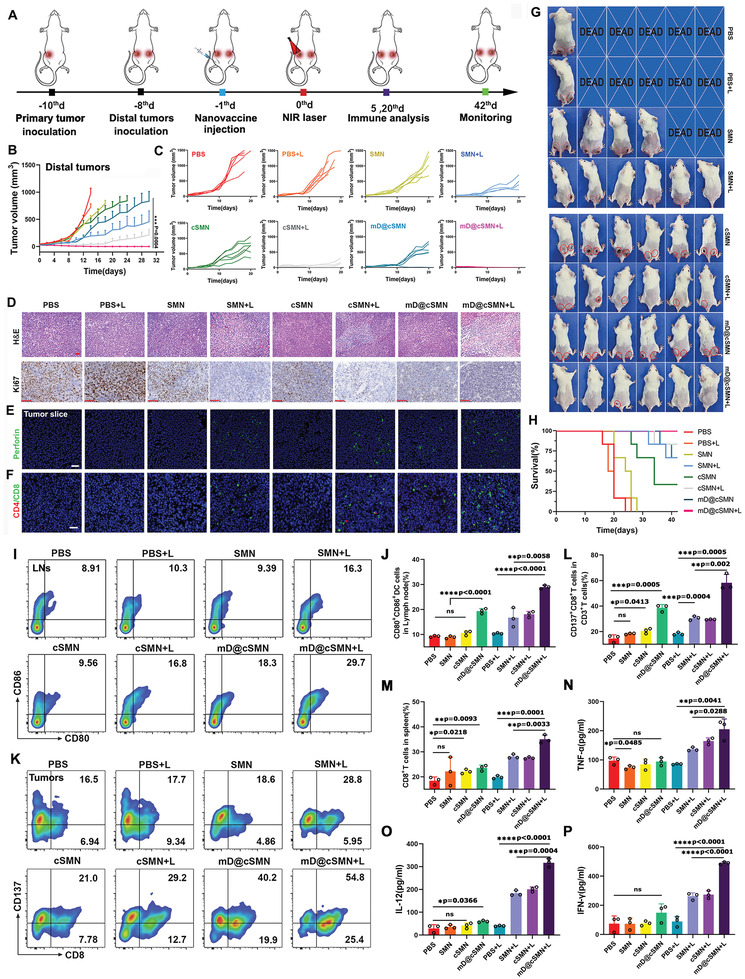
The synergistic antitumor effect of mD@cSMN nano‐vaccines inhibition of distal tumor growth and enhanced the CTLs infiltration and cytokine secretion. A) Schematic illustration of the process of s.c. injection of mD@cSMN nano‐vaccines in distal tumor mode. B,C) The average distal tumor volume change of mice after different treatments as indicated, (*n* = 6). D) H&E and ki67 staining of tumor slice at the 20th day after receiving different treatment as indicated, scale bar, 50 and 100 µm, respectively. CLSM image of perforin (E,F) tumor‐infiltrating lymphocytes (TILs) in the primary tumors at the 20th day after receiving different treatments as indicated. Red fluorescence is CD4+T cells. Green fluorescence is CD8+T cells, scale bar, 50 and 20 µm, respectively. G) Digital images of mice with inhibition of tumor growth in each group on 20th day after receiving different treatment as indicated (*n* = 6). H) Survival curves of the H22 tumor‐bearing mice (*n* = 6) after immunization and irradiated by NIR laser (0.1 W cm^−2^) for 5 min as indicated. I,J) Induced DC maturation in tumor‐draining lymph nodes after inoculation with PBS, SMNs, cSMNs, and mD@cSMNs with or without NIR laser irradiation. The immune cells in LNs are collected and analyzed by FACS after staining with CD11c, CD80, and CD86 on the 5th day, respectively, (*n* = 3). K–M) The TILs in tumors after receiving different treatments as indicated. The T cells in the tumor are collected and analyzed by FACS after staining with CD3, CD8, and CD137 on the 5th day, (*n* = 3). Cytokine levels of TNF‐*α* (N), IL‐12 (O), and IFN‐*γ* (P) in primary tumors isolated from differently treated mice by ELISA analysis, (*n* = 3). The statistical analysis is performed with ANOVA analysis, *p < 0.05, **p < 0.01, ***p < 0.001, ****p < 0.0001. Data are presented as mean ± SEM.

To further verify the functions of captopril, we assessed the different phenotypes of neutrophils in primary tumors by detecting the specific surface markers of CD11b and Ly6G. As shown in Figure 5G,J and Figures S24 and S25, Supporting Information, the percentage of protumoral N2 phenotype neutrophils (CD11b^high^ Ly6G^high^) in primary tumors was significantly reduced in mD@cSMN (32.1% ± 1.48%) or cSMN (31.63% ± 1.46%) treated groups, comparing to PBS (50.57% ± 0.65%) or SMN (47.3% ± 2.16%) treated groups without laser irradiation due to the capacity of released captopril.^[^ ^22^ ^]^ Similarly, upon the laser irradiation, the population of N2 phenotype neutrophils in mDCs@cSMN (26.9% ± 5.63%) or cSMN (25.67% ± 5.15%) treated groups were also obviously reduced compared to the PBS (51.4% ± 2.89%) or SMN treated groups (52.27% ± 2.89%) at the same conditions, indicating that the protumoral N2 phenotype neutrophils in tumors were efficiently reduced. Additionally, the splenic neutrophils showed similar results of the phenotypic marker expression of CD11b^low^ Ly6G^low^ in mDCs@cSMNs (4.26% ± 0.78%) or cSMN (4.49% ± 0.99%) treated groups comparing with the PBS (12.67% ± 3.42%) or SMN (11.77% ± 0.35%) treated groups at the same conditions, demonstrating the released captopril indeed could reduce the immunosuppressive N2 phenotype neutrophils. To verify the polarization of N2 phenotype neutrophils to N1 phenotype neutrophils, we assessed the level of CD95 and CD54 (as the typical N1 neutrophil surface markers^[^ ^43^, ^44^ ^]^) in neutrophils from tumors after mD@cSMN+NIR treatment by flow cytometry. As shown in **Figure** **6**K, the CD95 and CD54 markers were significantly increased in neutrophils from mD@cSMN +NIR treated tumors compared to the neutrophils from PBS treated tumors, implying the increase of N1 phenotype neutrophils; together with the results shown in Figure 5G–J, the decrease of N2 phenotype neutrophils after mD@cSMN +NIR treatment, we concluded that that the captoprils released from mD@cSMN indeed polarized the tumor‐associated neutrophils from N2 phenotype to N1 phenotype.

Besides, PDT‐induced ICD has been demonstrated to use as an in situ tumor vaccination to elicit the systemic antitumor immune responses to suppress the distal tumor growth through abscopal effect.^[^ ^31^, ^34^ ^]^ Thereafter, we thus investigated the tumor growth inhibition of distal tumor without direct treatment. As shown in Figure 6A–C, the SMN + NIR group showed certain growth inhibition of untreated distal tumor compared to the PBS + NIR group, demonstrating the abscopal effects triggered by PDT alone. But it still was insufficient to effectively control the distal tumor progress. Compared to the SMN + NIR group, the distal tumor growth was suppressed obviously in cSMN + NIR group, suggesting the synergistic effect of TAA related systemic antitumor immune response triggered by PDT and captopril functions; while the mD@cSMN + NIR showed the best tumor growth suppressive effect in the distal tumor, indicating the synergistic antitumor effect of TAA related systemic antitumor immune response triggered by PDT, the TSA specific immune response induced by mDC membrane from mD@cSMN and the captopril functions.

To further investigate the antitumor activity and corresponding mechanisms of mD@cSMNs, the ex vivo tumors from different treated groups were analyzed by H&E, immunohistochemistry analysis, and immunofluorescence, respectively. The H&E and ki67 (cell proliferation) images showed more serious cell apoptosis with extensively damaged areas and rare cell proliferation areas in the mD@cSMN + NIR group than that of PBS, SMN, or cSMN groups at the same conditions, suggesting an excellent synergistic antitumor effect of mD@cSMN (Figure 6D and Figure S26, Supporting Information). Additionally, a relatively high amount of green fluorescence from perforin was observed in mD@cSMN + NIR group compared to other treated groups at the same conditions, indicating an effective infiltration of CD8+T cells into tumors, and further verified by immunofluorescence with staining CD4 and CD8 antibodies (Figure 6E,F). To further verify the vital role of CD8+T cells in mD@cSMN+NIR group, we have depleted CD8+T cells by anti‐CD8 antibody. As shown in Figure S27, Supporting Information, after intraperitoneal injection of anti‐CD8 antibody (5 mg mL^−1^) to mice, the tumor inhibition efficiency of mD@cSMN+NIR group after depletion of CD8+T cells was significantly lower than that of mD@cSMN+NIR treatment without T cell depletion, indicating the vital role of CD8+T cells in antitumor immune response. Furthermore, to directly visualize the synergistic antitumor effect, the mice that received different treatments as indicated on the 20th day were imaged by a digital camera (*n* = 6). As shown in Figure 6G, 5/6 of mice with excellent suppression of tumor growth were observed in mD@cSMN + NIR group both in the primary and distal tumors versus PBS+ NIR (0/6), SMN+ NIR (3/6), and cSMN+ NIR (3/6) groups. After observation for 40 days, the 6/6 of mice with complete prevention of primary and distal tumor growth were achieved after being treated with mD@cSMNs + NIR as compared with PBS + NIR (0/6), SMN + NIR (1/6), and cSMN + NIR (1/6) groups, respectively (Figure 5H). These findings clearly demonstrated that the combination of PDT and captopril with nano‐vaccines could effectively convert H22 tumor immune “cold” state into “hot” to boost strong systemic antitumor immune responses for synergistically suppressing tumor growth.

To elucidate the immune mechanisms of synergistic therapeutic efficiency by mD@cSMNs to inhibit the primary tumors and distal tumors through their abscopal effects in vivo, we first investigated the maturation of DCs in tumor‐draining LNs after receiving different treatments since the matured DCs enable to present the antigens and stimulate T‐Cell responses.^[^ ^19^ ^]^ The mDCs in LNs were evaluated by FACS with staining the co‐stimulatory molecules of CD11c, CD80, and CD86 antibodies. As shown in Figure 6I,J, and S28, the matured DC population (CD80+CD86+) showed an increase in SMN + NIR group (16.73% ± 3.87%) or cSMN + NIR group (18.07% ± 1.17%) compared to the PBS + NIR group (9.27% ± 0.33%) in primary tumors, demonstrating that the PDT‐induced ICD could stimulate the DC maturation. While the matured DC population was further significantly increased in mD@cSMN + NIR group (28.97% ± 0.75%) than that of any other groups mainly attributing to combination of both TAA and TSA stimulations. Because of the CD137+(4‐1BB)CD8+ co‐expressed T cells as activated T cells with efficient anti‐tumor abilities,^[^ ^45^ ^]^ thereafter, we checked the activated CD137+CD8+T cell numbers in tumors after treatments since the matured DC enable to potentiate the activation of and infiltration of CD8+T cells.^[^ ^20^ ^]^ As shown in Figure 6K–M and Figures S25 and S29–S31, Supporting Information, the percentage of CD137+ CD8+T cells infiltrated in primary tumors was significantly increased in mD@cSMNs treated group alone (38.53% ± 2.72%), compared to PBS (14.6% ± 2.87%), SMNs (18.3% ± 0.52%), or cSMNs (20.53% ± 1.94%) treated groups without laser irradiation attributing to the efficient activation of CD8+T cells by mD@cSMNs, resulting in the enhancement of CD8+T cell infiltration into the primary tumors. More strikingly, with the help of PDT treatment to break the compact TME for improving activated T cell infiltration, the population of CD137+CD8+ activated T cells in primary tumors was further significantly increased in all treated groups including mD@cSMNs treated group (58.3% ± 26.5%), SMN treated group (30.53% ± 21.62%), and cSMN treated group (29.46% ± 0.25%), but still, the mD@cSMNs treated group showed the highest percentage of CD137+CD8+ activated T cells. Meanwhile, a significantly higher level of TNF*α*, IL‐12, and IFN*γ* in primary tumors were clearly observed in mD@cSMN + NIR group compared to other groups, which mainly attributed to both of TAA and TSA activation of systemic immune responses to result in the infiltration of TILs in the primary tumors (Figure 6N–P). Furthermore, we checked the T_CM_ and T_EM_ cell populations in tumor‐draining LNs of mice after mD@cSMN+NIR treatment. As shown in Figure S32, Supporting Information, compared to the PBS treated group, the mD@cSMN+NIR treated group showed higher frequency of CD8+T_EM_ cells while the relative lower frequency of CD8+T_CM_ cells, suggesting the ability of this therapy to provide a more potent antitumor immune memory response to protect against distal tumor growth. Besides, to further assess the H22 specific antitumor effect, we inoculated H22 tumor cells and 4T1 breast cancer cells into the back of mice in left and right sides. After injection of mD@cSMN for 24 h, the H22 tumor was then irradiated by NIR laser for 5 min. The 4T1 tumor volume was then evaluated by vernier caliper. The PBS‐treated group was taken as the control. As shown in Figure S33, Supporting Information, we clearly saw that a rapid tumor growth of 4T1 tumor was observed after mD@cMSN treatment with NIR irradiation, which was similar to the PBS treated group. While the distal tumor growth of H22 bearing mice without NIR laser irradiation in Figure 6B was significantly inhibited, indicating the H22 specific antitumor effect. These findings suggested that the mD@cSMN combined with laser irradiation could effectively convert H22 tumors from “cold” to “hot” to induce robust systemic anticancer H22‐specific T cells immune responses against primary and distal tumors. Besides, we found that the H22 tumor‐bearing mice with inoculation of mD@cSMNs followed by laser irradiation had no significant body weight loss and major organs damages (H&E staining) (Figures S34 and S35, Supporting Information), indicating the low systematic toxicity. Overall, mD@cSMN nano‐vaccines could actively target tumors, home to LNs, and directly activate T cell immunity, reduce the protumoral N2 phenotype TANs to further improve the immune effects for synergistic inhibition of both the primary and distal HCC tumor growth, which might provide a promising therapeutic vaccination strategy for potentiating HCC immunotherapy.

## 3 Conclusion

In summary, an acidic/photo‐sensitive DC‐based neoantigen nano‐vaccine has been designed and prepared to efficiently convert tumor immune “cold” state into “hot”, through potentiate TAA/TSA specific T cell immune responses and remodel tumor‐associated neutrophils for potentiating immunotherapy efficiency of HCC. In our design, the SiPCCl_2_‐hybridized mesoporous silica (SMNs) with excellent water stability and photo‐stability acted as a new nano photosensitizer, and its inherent porous nanostructure has been loaded with captopril through Fe(III) mediated coordination effect. After coating with H22‐specific neoantigen activated DC membrane, it not only enables to actively target to tumors for efficient PDT to enhance ICDs for in situ vaccination to strengthen TAA related T‐cell response, but also possess an excellent lymph‐homing effect to directly induce the activation and proliferation of TSA‐specific CD8+T cell immunity to suppress both the primary tumor and distal tumor growth. More strikingly, the protumoral N2 phenotype tumor‐associated neutrophils can be obviously reduced through TME acidic‐triggered captopril release to further augment the immune effects for synergetically inhibiting tumor growth, leading to prolonged survival of H22‐bearing mice. Taking together, this novel therapeutic vaccination strategy might provide valuable insight for designing personalized cancer vaccines for HCC immunotherapy.

## 4 Experimental Section

### Materials

Silicon phthalocyanine dichloride (SiPCCl_2_), 3‐aminopropyltrimethoxysilane (APTES), Hoechst 33342, tetraethylorthosilicate (TEOS), and 2, 7‐dichlorodihydroflfluorescein diacetate (DCFH‐DA) were obtained from Sigma‐Aldrich. Hexadecyl trimethylammonium chloride (CTAC), triethanolamine (TEA), and FeCl_3_ were obtained from Sinopharm Chemical Reagent Co. Ltd. Dylight550‐NHS was purchased from Thermo Fisher Scientific (Waltham, USA). ICG‐NHS was obtained from Xi'an Rixi Biological Technology Co. Ltd (Xi'an, China). Calcein‐AM/propidium iodide (PI), and 1, 3‐diphenylisobenzofuran (DPBF) were obtained from KeyGen BioTech (Shanghai, China). Annexin V‐FITC/PI and CCK8 were obtained from Dojindo Laboratories. Annexin V‐APC/PI was obtained from Jiangsu KeyGEN BioTECH Corp., Ltd (Jiangsu, China) Neutrophil (mouse) isolation Kit was purchased from Cayman Chemical (Michigan, USA). Anti‐CD11c‐APC, anti‐CD137‐APC, anti‐CD11b‐APC, anti‐CD86‐PE‐Cy7, antiCD3‐APC, anti‐CD80‐PE, anti‐CD8‐PE, anti‐Ly‐6G/Ly‐6C‐PE, CD54, CD95, anti‐CD8 antibody, and anti‐CD69‐FITC was purchased from BioLegend, Inc (San Diego, CA, USA). ELISA kits from Neobioscience Technology (Shenzhen, China) were obtained to detect the IFN‐*γ*, TNF‐*α*, IL‐12, and IL‐2. H22 mouse liver cancer cell‐specific neoantigen (sequence: HTDAHAQAFAALFDSMH) was obtained from GenScript USA Inc.

### Cell Culture

H22 cells (mouse liver cancer) were cultured at 37 °C in the incubator with RPMI‐1640 medium (100 IU mL^−1^ penicillin‐streptomycin and 10% FBS) BNL CL2 mouse embryonic hepatocyte cells were cultured at 37 °C in the incubator with Dulbecco's modified eagle medium (100 IU mL^−1^ penicillin‐streptomycin and contain 10% FBS, Cellgro, Manassas, VA, USA).

### Synthesis of mD@cSMN

To obtain the mD@cSMN nano‐vaccines, the SMN was synthesized with the previously reported method.^[^ ^40^ ^]^ Briefly, 3 mg of SiPCCl_2_ was first dissolved in EtOH and then mixed with an aqueous solution containing CTAC (2 g) and TEA (20 mg) with vigorous stirring at 80 °C for 1 h. Afterward, 1.5 mL of TEOS was slowly dropped into the above mixture with vigorous stirring for another 1 h. After cooling, the obtained products were centrifuged and washed by methanol containing 1 wt% sodium chloride 3 times to remove CTAC. Subsequently, the obtained SMN was mixed with APTES (60 µL) in ethanol and refluxed at 40 °C for 4 h with vigorous stirring. The obtained SMN‐NH_2_ was purified by ethanol, dissolved in deionized water, and then mixed with FeCl_3_ (1 mg mL^−1^) for 2 h. Afterward, the captopril (5 mg mL^−1^) was added with stirring for 24 h. The obtained cSMN was centrifuged and washed with deionized water 3 times. The captopril in cSMN was quantified by high‐performance liquid chromatography (HPLC) (Agilent 1260 Infinity, Agilent Technologies, Germany) through using Agilentzorbax Eclipes Plus C18 Colum (4.6 mm × 100 mm, 3.5 µm, Agilent Technologies, USA) by isocratic elution with 60% of methanol and 40% of 0.05% phosphoric acid. The column temperature was kept at 22 °C, and the flow‐rate was maintained at 1 mL min^−1^. The correlation between the peak area at 1.5 min, and the concentration of captopril was quantified according to the linear (*Y* = 5133.3*x* + 67.651, *R* ^2^ = 0.999), the correlation curve was prepared from 0–1.25 mg mL^−1^. Finally, the immature mouse bone marrow‐derived cells were co‐incubated with H22 specific‐neoantigens for 2 days, and then examined by flow cytometry (FACS) with staining anti‐CD11c‐APC, anti‐CD80‐PE, and anti‐CD86‐PE‐Cy7 antibodies. Afterward, the membrane of matured mDCs was extracted through the manufacturer's protocol and then mixed with cSMNs at the mass ratio of 1:1 with sonication for 15 min in the ice bath. The mD@cSMN was then obtained and purified by centrifugation.

### Characterization

To prove the successful coating on cSMN with mDCs membrane, mD@cSMNs, and the purified mDCs membrane were prepared in 1× loading buffer solution with ratio of 1:1 through the BCA kit ration. Then, the samples containing mD@cSMNs or mDCs membrane were loaded onto sodium dodecyl sulfate‐polyacrylamide gel electrophoresis gel (10%), ranging at 100 V for 1.5 h. In addition, these samples were also analyzed by immunoblotting using anti‐CD80, anti‐CD86, and anti‐MHC‐II antibodies at 4 °C, and subsequently incubating of HRP‐labeled secondary antibody (goat anti‐rabbit IgG, 1:5000) and co‐incubated for 1 h, which then were analyzed by ChemiDocTM MP imaging system.

To investigate the TME acidic triggered captopril release from mD@cSMN, the mD@cSMN was dispersed in PBS (pH 5.5 and 7.4). At the predetermined time intervals, 0.2 mL of the supernatant was withdrawn to assess the released amount of captopril through HPLC analysis after centrifugation. Moreover, to maintain a volume constantly, 0.2 mL of PBS with the corresponding acidic solution was supplemented to above after calculation.

### Detection of ROS Generation In Vitro

To assess the ROS generation of SMN, 10 µg mL^−1^ of SMN containing DPBF (8 mM) in DMF was irradiated by 670 nm laser with the power intensity of 50 mW cm^−2^. The absorbance (300 to 600 nm) and absorption peak of DPBF at 420 nm was recorded through a UV‐Vis‐NIR spectrograph (Spectro Max M5e, Germany).

To detect the ROS generation from H22 cells treated with mD@cSMNs, DCFH‐DA probes were used as the ROS fluorescence indicator. Briefly, 1 × 10^4^ per well of H22 cells were seeded in 96‐well plates. Afterward, mD@cSMNs (80 µg mL^−1^) were added and co‐incubated for 24 h. Then, the treated cells were stained by DCFH‐DA (50 µM) for 30 min and washed by PBS buffer, followed by 670 nm laser irradiation (50 mW cm^−2^) for 5 min. Finally, the treated cells were immediately analyzed by fluorescence microscopy (excitation at 488 nm).

### Cellular Uptake of mD@cSMN and the Phototoxicity to H22 Cells

The cellular uptake of mD@cSMNs via H22 cells was evaluated by confocal microscopy (LSM 780, Germany). Briefly, 2 × 10^5^ H22 cells were added into 35 mm glass‐bottom Petri dishes for 24 h at 37 °C. Then, DyLight550 NHS‐labeled mD@cSMNs (80 µg mL^−1^) with the fresh medium was added to the above H22 cells and co‐incubating for another 24 h. Afterward, the treated H22 cells were washed by PBS 2 times, and subsequently stained by Hoechst 33342 and dropped on the glass slide for CLSM imaging.

To investigate the cytotoxicity or phototoxicity of mD@cSMNs, the BNL CL2 mouse embryonic hepatocyte cells and H22 mouse liver cancer cells were used. Briefly, the BNL CL2 cells were seeded in 96‐well plates (2 × 10^4^ cells per well) and incubated at 37 °C for 24 h. Then, the cells were washed by PBS buffer 2 times, followed by incubation with a fresh culture medium containing various concentrations of mD@cSMNs for 24 or 48 h; Besides, the H22 cells (2 × 10^5^) were seeded onto 96‐well plates (2 × 10^4^ cells per well) and incubated for 24 h. Then, the fresh culture medium containing various concentrations of mD@cSMNs was co‐incubated for 24 h and then exposed to a 670 nm laser with the power intensity of 50 mW cm^−2^ for 5 min. After 24 h, the cytotoxicity or phototoxicity was analyzed through CCK8 kit.^[^ ^40^, ^41^ ^]^ Additionally, the antitumor efficacy of mD@cSMNs was investigated by a live/dead viability/cytotoxicity kit staining (Calcein‐AM/PI) and Annexin V‐APC/PI assay.^[^ ^29^ ^]^

### Photodynamic Efficiency and their ICD Effect of mD@cSMNs in DCs Stimulation

Tumor cell surface‐exposed CRT, actively secreted ATP, and released high‐mobility group box 1 protein (HMGB1) were considered as the DAMPs of ICD.^[^ ^34^, ^41^ ^]^ To evaluate the CRT, 2 × 10^4^ H22 liver cancer cells were seeded in 96‐well plates and cultured for 24 h. After being co‐incubated with mD@cSMNs for 24 h, the treated cells were irradiated by 670 nm lasers (50 mW cm^−2^) for 5 min. Afterward, the cells were collected and blocked by 3% BSA for 15 min. Afterward, the treated cells were incubated with CRT primary antibody (1:100) for 90 min. After washing by PBS buffer, the treated cells were incubated with anti‐rabbit IgG (Alexa Fluor 488) (1:500) for 60 min, and then analyzed by FACS.

To analyze the HMGB1 release, the treated cells after receiving above‐mentioned treatment were collected and treated with 4% paraformaldehyde for 25 min. Then, the treated cells were permeated with 0.3% Triton X‐100 for 20 min. By blocking through 3% BSA for 15 min, the treated cells were then co‐incubated with HMGB1 antibody (1:250) at room temperature for 90 min, and then washed once with PBS. Subsequently, the cells were treated with goat anti‐rabbit IgG (Alexa Fluor 488) (1:500) for 60 min and detected by FACS; besides, to detect the ATP, the treated H22 cells were followed by above‐mentioned process, and the ATP from treated cells were then detected by ATP Assay Kit through the manufacture's protocol. To further investigate the DC maturation, the H22 cells after receiving above‐mentioned different treatments were added into immature BMDCs, and cultured for another 48 h. Afterward, the BMDCs were then stained by anti‐CD80‐PE, anti‐CD11c‐APC, and anti‐CD86‐PE‐Cy7 antibodies, and detected through FACS.

### Nano‐Vaccine Induced T cell Activation, Proliferation, and Cytotoxicity to H22 cells

To assess the T cell activation, T cells were extracted from the spleen of BALB/c mice (6 weeks, female) by CD3 MicroBeads Kit through the manufacturer's protocols. Then, the obtained T cells were seeded in 24‐wells plates (5 × 10^5^ cells per well), and co‐incubated with mD@cSMNs (80 µg mL^−1^) for 36 h without NIR laser irradiation. Then, the T cells were stained by anti‐CD69‐FITC antibody and analyzed by FACS. To examine the T cell proliferation, 1 × 10^4^ T cells were added into 96‐wells plates and then stained with CFSE fluorescence. Afterward, the CFSE‐labeled T cells were co‐incubated with mD@cSMNs (80 µg mL^−1^) for 3 days and subsequently analyzed by FACS.

To evaluate the cytotoxicity of activated T cells to H22 cancer cells, the T cells were added to 24‐wells plates (2 × 10^5^ cells per well) with IL‐2 (10 ng mL^−1^), and then were co‐incubated with mD@cSMNs for another 48 h for T cell activation and proliferation. Besides, the activated T cells were then co‐incubated with H22 cells for 36 h, and followed by staining with anti‐CD3‐APC antibodies and the treated H22 cells were stained with Annexin V‐FITC and PI. Subsequently, the cytotoxicity of activated T cells to H22 cancer cells was evaluated by FACS. Besides, the supernatant was collected and then detected by ELISA kit (TNF‐*α*, IFN‐*γ*, and IL‐2) through the manufacturer's instruction.

### In Vivo Fluorescence Imaging, Immunoassay, and Antitumor Efficiency of mD@cSMNs

First, to investigate the targeting ability of mD@cSMNs, the BALB/c mice (6 weeks, female, China Wushi, Inc. Shanghai, China) were s.c. injection of H22 cells (3 × 10^6^ cells) in PBS solution. All animal procedures were conducted according to the “National animal management regulations of China” and approved through the Animal Ethics Committee of Mengchao Hepatobiliary Hospital of Fujian Medical University. When the tumor size reached about 100 mm^3^, the mice were subcutaneously inoculated with ICG‐NHS labeled SMNs or mD@cSMN nano‐vaccines. After 24 h, the mice were sacrificed. The lymph nodes, tumors, and major organs including the heart, spleen, kidney, lung, and liver were obtained and imaged by UniNano NIR‐II imaging system. To evaluate the antitumor effects, the BALB/c mice (6 weeks, female) were s.c. injection of H22 cancer cells (3 × 10^6^). When the tumor size reached ≈100 mm^3^, the H22‐bearing mice were randomly divided to 8 groups by subcutaneous inoculation as follow:

- Sterilized PBS buffer without Laser (*n* = 12);
- Sterilized PBS buffer with Laser (0.1 W cm^−2^) for 5 min (*n* = 12);
- SMN (1 mg mL^−1^) without Laser (n = 12);
- SMN (1 mg mL^−1^) with Laser (0.1 W cm^−2^) for 5 min (*n* = 12);
- cSMN (SMN, 1 mg mL^−1^; captopril, 0.2 mg mL^−1^) without Laser (*n* = 12);
- cSMN (SMN, 1 mg mL^−1^; captopril, 0.2 mg mL^−1^) with Laser (0.1 W cm^−2^) for 5 min (*n* = 12);
- mD@cSMN (SMN, 1 mg mL^−1^; captopril, 0.2 mg mL^−1^) without Laser (*n* = 12);
- mD@cSMN (SMN, 1 mg mL^−1^; captopril, 0.2 mg mL^−1^) with Laser (0.1 W cm^−2^) for 5 min (*n* = 12).

The tumor volume was recorded through electronic vernier caliper every two days. The mice weight was measured every two days. The tumor volume (*V*) was calculated through blow formula^[^ ^41^ ^]^:

(1) $$V_{\mathrm{volume}}={\left({\mathrm{Long}}_{\mathrm{tumor}\;(\mathrm{mm})}\times {\mathrm{width}}_{\mathrm{tumor}\;(\mathrm{mm})}\right)}^{2}/2\in$$

To assess the immune responses, the H22 tumor‐bearing mice received various treatments were sacrificed on the 5th day (*n* = 3). The lymph nodes were isolated. DC cells from lymph nodes were harvested and examined by FACS with staining anti‐CD11c‐APC, anti‐CD80‐PE, and anti‐CD86‐PE‐Cy7 antibodies, respectively. In addition, to evaluate the activation of TILs, the tumors from mice that received different treatments were isolated and prepared for cell suspension according to the previously published works.^[^ ^42^ ^]^ Afterward, the cells were harvested and examined by FACS with staining anti‐CD3‐FITC, anti‐CD8‐PE, and anti‐CD137‐APC antibodies, respectively. Moreover, to analyze the T cell activation in mouse spleen, CD8+T cells were isolated from mice received different treatments as indicated according to the previously published works.^[^ ^41^ ^]^ Afterward, the T cells in spleens were harvested and analyzed by FACS with staining anti‐CD3 ‐APC and anti‐CD8‐PE antibodies. The cytokine (TNF*α*, IFN*γ*, and IL‐12) from tumors were also detected by using Mouse ELISA Kit according to the manufacturer's instruction.

To investigate the state of neutrophils, the tumor and spleen from different treated mice were isolated on the 20th day (*n* = 3). Then, the neutrophil was isolated from tumors or spleen according to the manufacturer's instruction. Afterward, the cell was harvested and analyzed by FACS with staining anti‐CD11b‐APC and anti‐Ly‐6G/Ly‐6C‐PE antibodies. In addition, the neutrophils from tumors were extracted by Neutrophil (mouse) isolation Kit and then stained with CD54 and CD95 antibodies, and co‐incubated with secondary antibodies for flow cytometry analysis.

### Immunofluorescence Assays and Pathological Changes

To analyze the expression of perforin in H22 tumor tissues, the tumors from different treated mice were isolated on the 5th day, and their tumor slices were then stained with anti‐perforin antibody for 1 h after blockage of tumor slices through serum for 10 min. Afterward, the tumor slices were treated with Alexa Fluor 488 labeled anti‐rabbit IgG (1:500) for 30 min, and then washing by PBS 2 times. The stained tumor slices were imaged by CLSM. In addition, to further assess the pathology change of tumors after receiving different treatments, the tumor slices were stained by H&E and immunohistochemical staining through Ki67. Furthermore, to assess the long‐term systematic toxicity of mD@cSMNs, the treated mice were sacrificed (20th day), and the heart, spleen, kidney, lung, and liver were then collected and treated with 10% paraformaldehyde, followed by slicing and staining with hematoxylin and eosin, and then recorded by microscopy.

### Statistical Analysis

The statistical analysis of all data was performed using the two‐tail paired Student's t‐test analysis for comparison between 2 groups or one‐way analysis of variance (ANOVA) for comparison among multiple groups through the GraphPad Prism 8.0 software. Survival curves were generated using Kaplan‐Meier estimates and tested using the log‐rank test. The *p < 0.05 was representing to statistically significant. **p < 0.01, ***p < 0.001, ****p < 0.0001. All numerical data are presented as the means ± SEM through at least three experiments.

## Conflict of Interest

The authors declare no conflict of interest.

## Supporting information

Supporting Information

## Data Availability

Research data are not shared.
