# Supplementary material for: Remodeling Tumor‐Associated Neutrophils to Enhance Dendritic Cell‐Based HCC Neoantigen Nano‐Vaccine Efficiency
Source: Adv Sci (Weinh). 2022 Feb 10;9(11):2105631. doi: 10.1002/advs.202105631 (PMC9009112; doi:10.1002/advs.202105631)
Supplement: Supplementary file 1 — Supporting Information [file ADVS-9-2105631-s001.pdf]

ADVANCED SCIENCE
Open Access

Supporting Information

for *Adv. Sci.*, DOI 10.1002/advs.202105631

Remodeling Tumor-Associated Neutrophils to Enhance Dendritic Cell-Based HCC Neoantigen Nano-Vaccine Efficiency

Yunhao Wang, Qingfu Zhao, Binyu Zhao, Youshi Zheng, Qiuyu Zhuang, Naishun Liao, Peiyuan Wang, Zhixiong Cai, Da Zhang, Yongyi Zeng* and Xiaolong Liu**

ADVANCED SCIENCE
Open Access

Supporting Information

for *Adv. Sci.*, DOI: 10.1002/advs.202105631

Remodeling Tumor-Associated Neutrophils to Enhance Dendritic Cell-Based HCC Neoantigen Nano-vaccine efficiency

Yunhao Wang, Qingfu Zhao, Binyu Zhao, Youshi Zheng, Qiuyu Zhuang, Naishun Liao, Peiyuan Wang, Zhixiong Cai, Da Zhang,Yongyi Zeng*, and Xiaolong Liu**

Supporting Information

Remodeling Tumor-Associated Neutrophils to Enhance Dendritic Cell-Based HCC Neoantigen Nano-vaccine efficiency

Yunhao Wang[1†], Qingfu Zhao[1†], Binyu Zhao[1], Youshi Zheng[1,4], Qiuyu Zhuang[1,4], Naishun Liao[1,4], Peiyuan Wang[3], Zhixiong Cai[1,4], Da Zhang[1,4]*, Yongyi Zeng[1,2]*, Xiaolong Liu[1,3,4]*

Y. Wang, Q. Zhao, B. Zhao, Y. Zheng, N. Liao, Prof. Z. Cai, Prof. D. Zhang, Prof. Y. Zeng, Prof. X. Liu
[1]The United Innovation of Mengchao Hepatobiliary Technology Key Laboratory of Fujian Province, Mengchao Hepatobiliary Hospital of Fujian Medical University, Fuzhou 350025, P. R. China
Prof. Y. Zeng,
[2]Liver Disease Center, The First Affiliated Hospital of Fujian Medical University, Fuzhou 350005, People's Republic of China
Prof. P. Wang, Prof. X. Liu,
[3]CAS Key Laboratory of Design and Assembly of Functional Nanostructures, Fujian Institute of Research on the Structure of Matter, Chinese Academy of Sciences, Fuzhou 350002, P. R. China
Y. Zheng, Prof. Q. Zhuang, N. Liao, Prof. Z. Cai, Prof. D. Zhang, Prof. X. Liu,
[4]Mengchao Med-X Center, Fuzhou University, Fuzhou 350116, P. R. China
*E-mail: zdluoman1987@163.com; lamp197311@126.com; xiaoloong.liu@gmail.com;

[†]These authors contributed equally to this work.

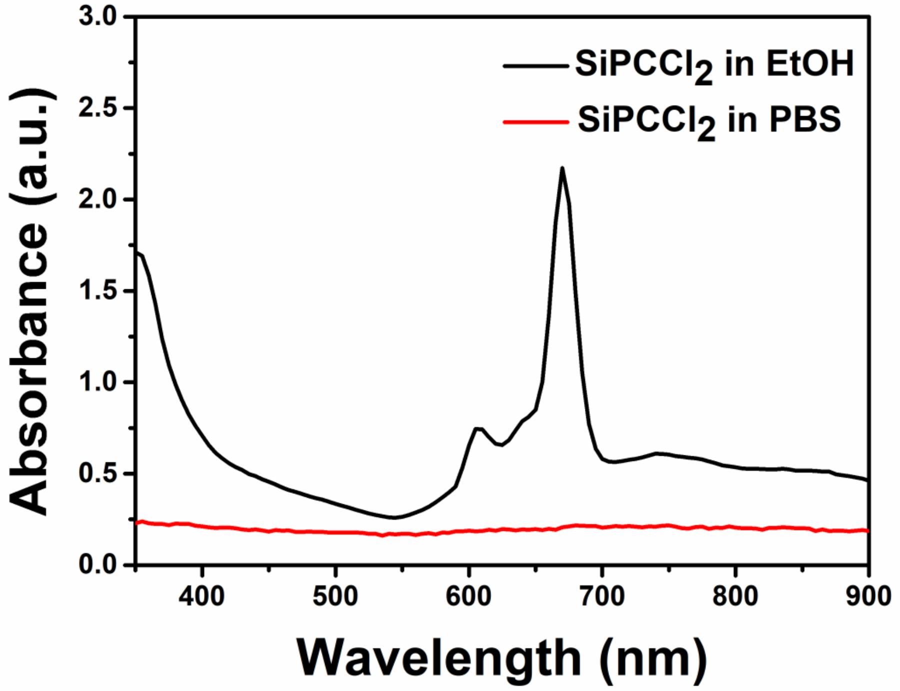

Figure. S1. The UV-vis-NIR absorbance of $SiPCCl_2$ in EtOH and PBS buffer.

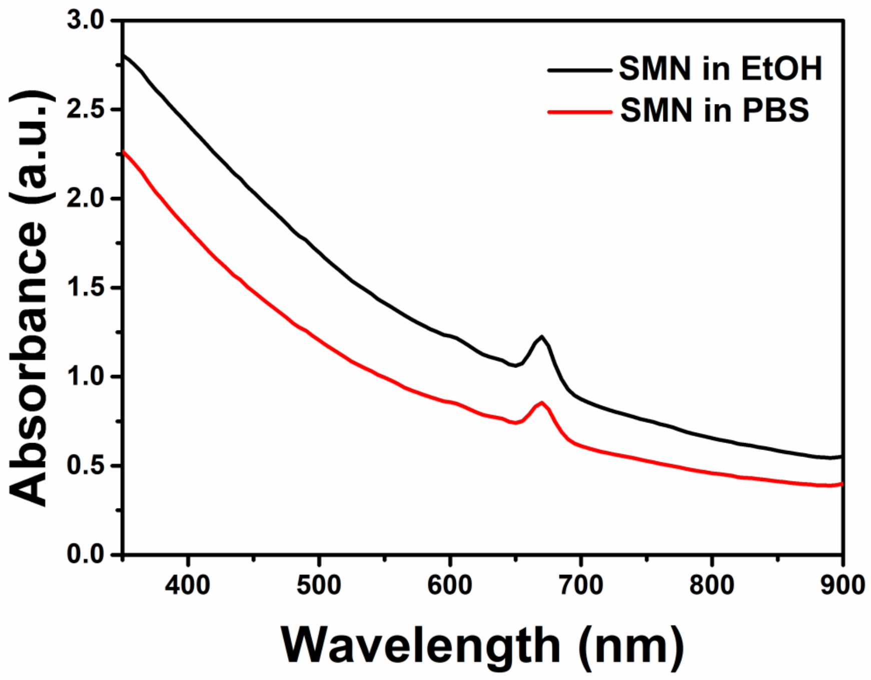

Figure. S2. The UV-vis-NIR absorbance of SMN nanophotosensitizer in EtOH and PBS buffer.

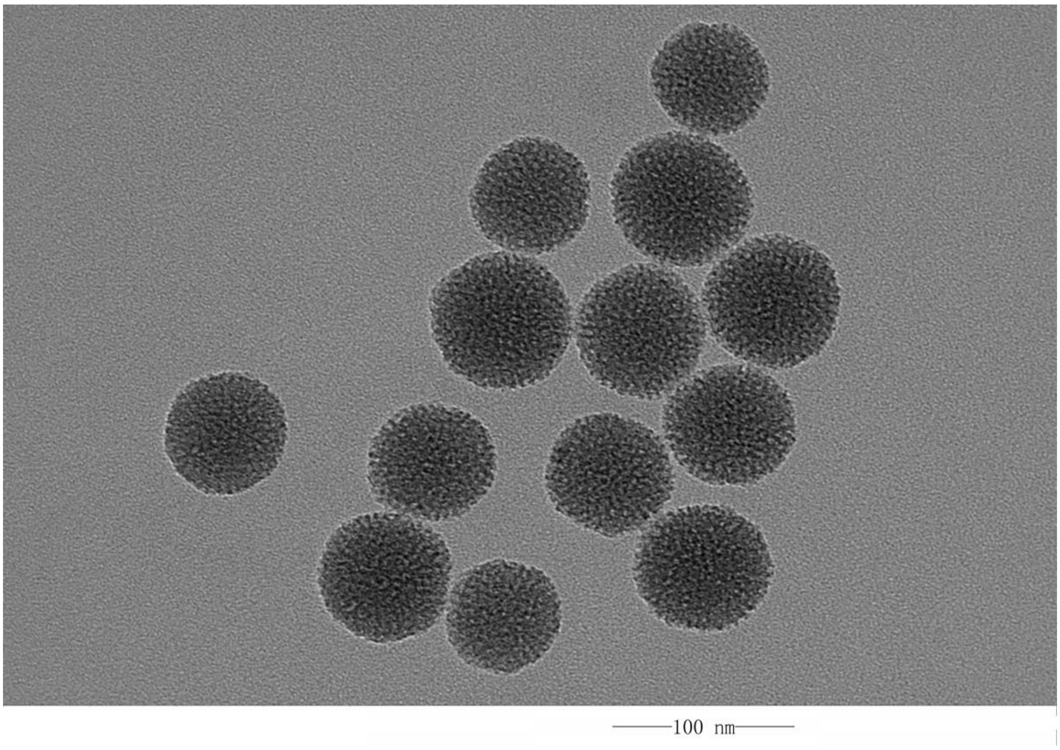

Figure. S3. Original TEM image of SMN nanophotosensitizer.

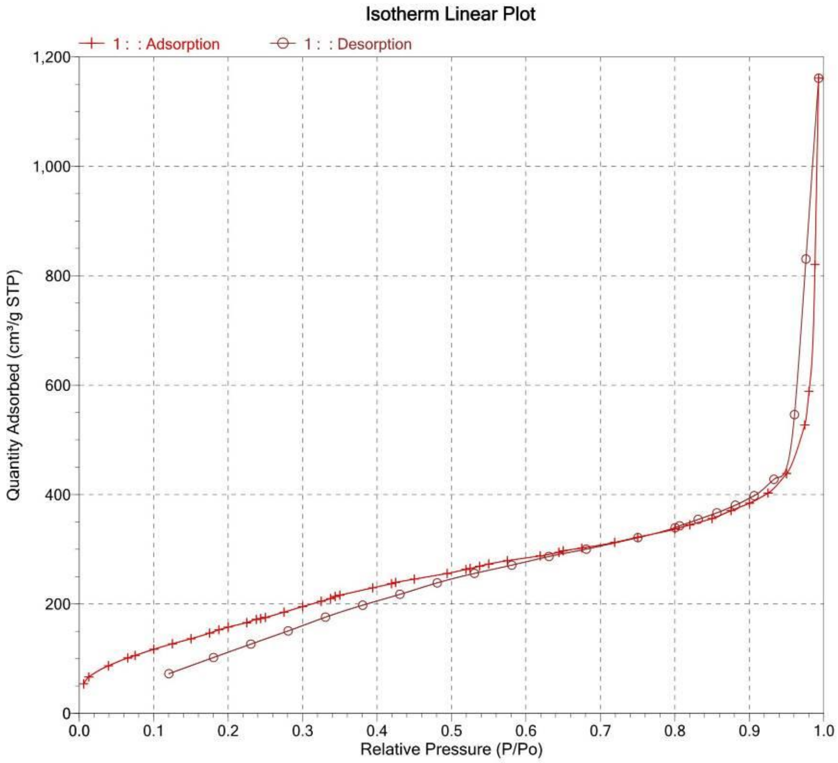

Figure. S4. N_2 sorption isotherm for SMN nanophotosensitizers.

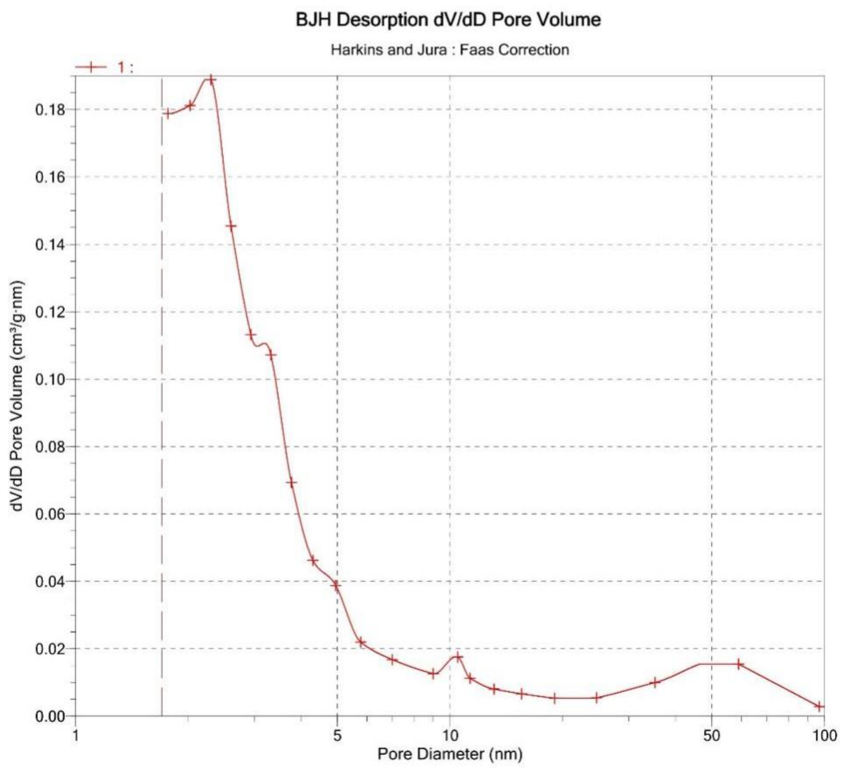

Figure. S5. DFT porous size distribution for SMN nanophotosensitizers.

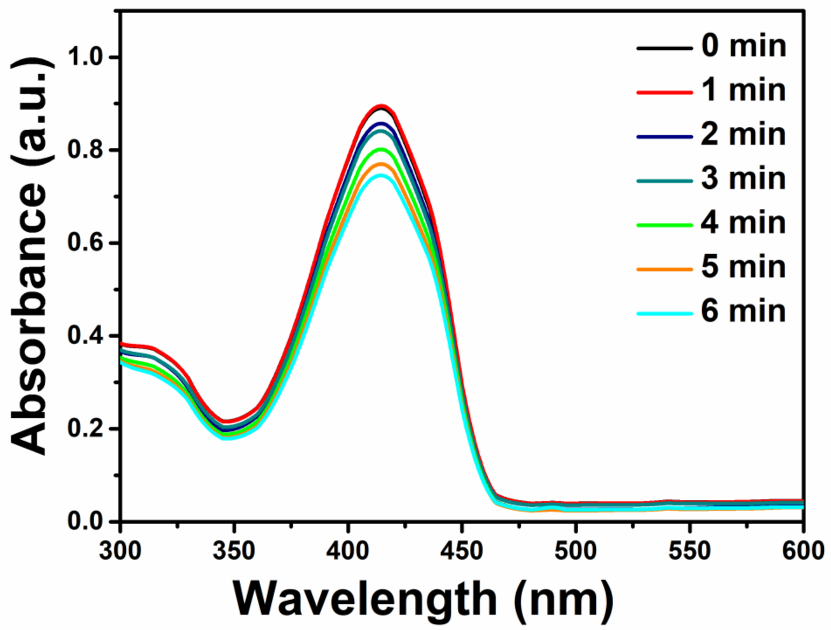

Figure. S6. The absorbance of DPBF with 670 nm laser irradiation (50 mW/cm^2) for different times.

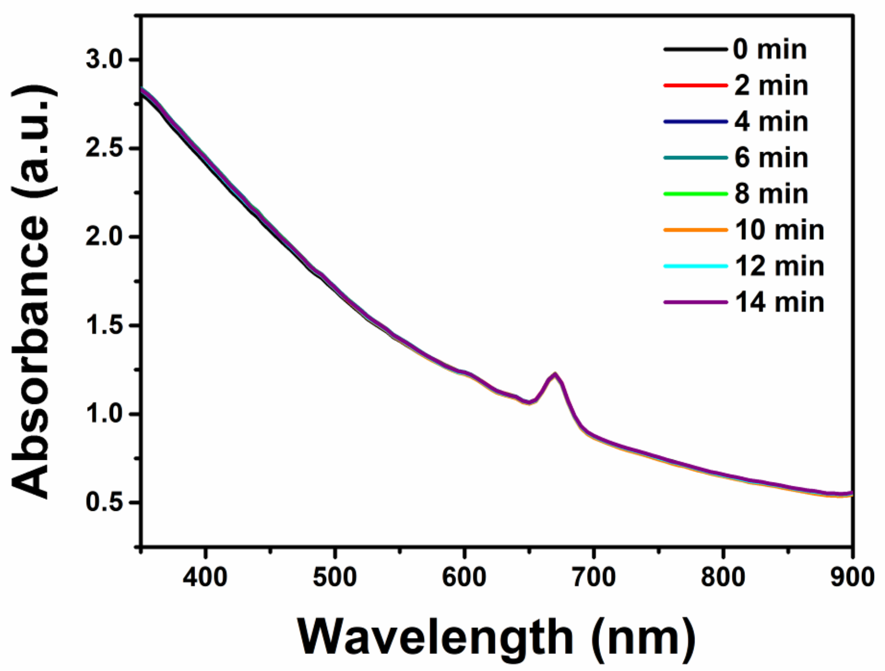

Figure. S7. The photostability of SMN in PBS buffer after 670 nm laser irradiation (50 mW/cm^2) for different times.

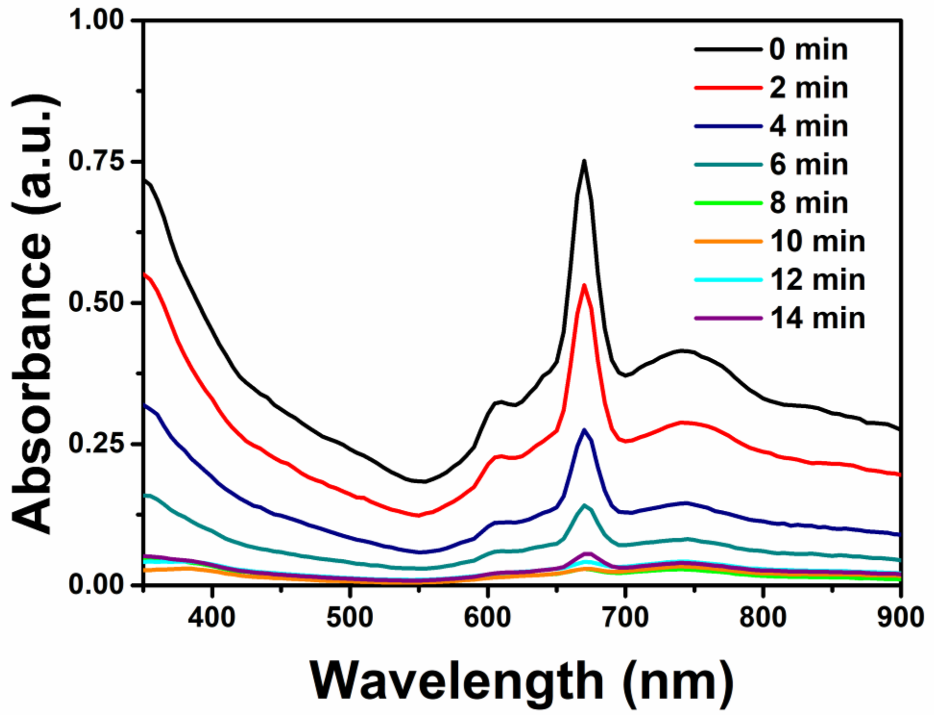

Figure. S8. The photostability of $SiPCCl_2$ in EtOH after 670 nm laser irradiation (50 mW/cm^2) for different times.

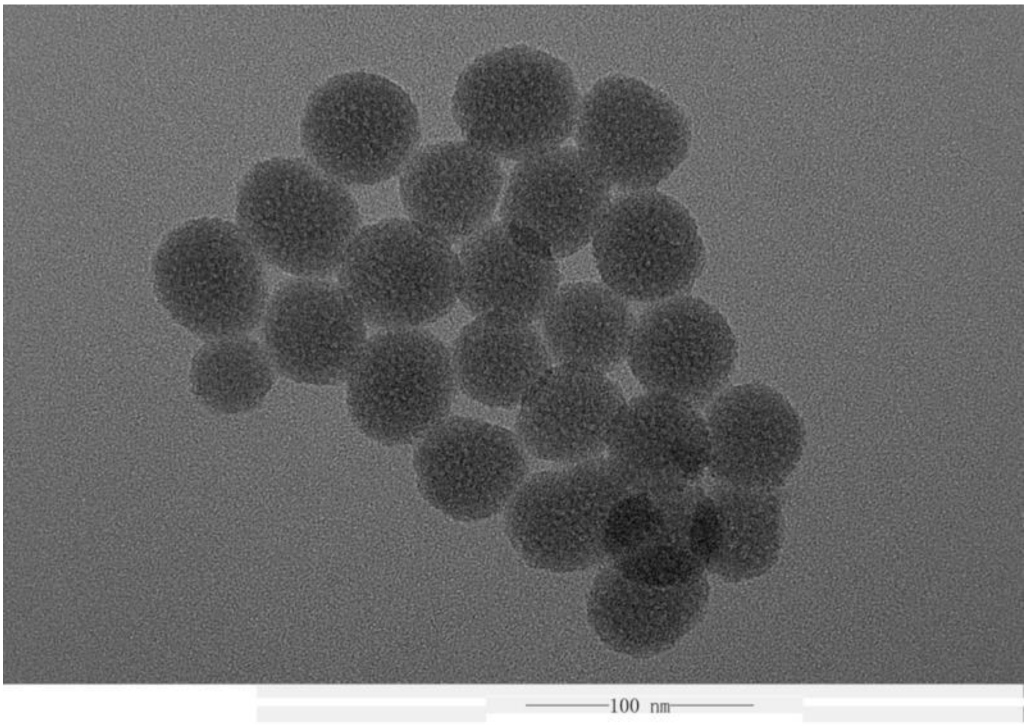

Figure. S9. The orgainal TEM image of cSMNs (SMN loaded with Fe(III)-captopril).

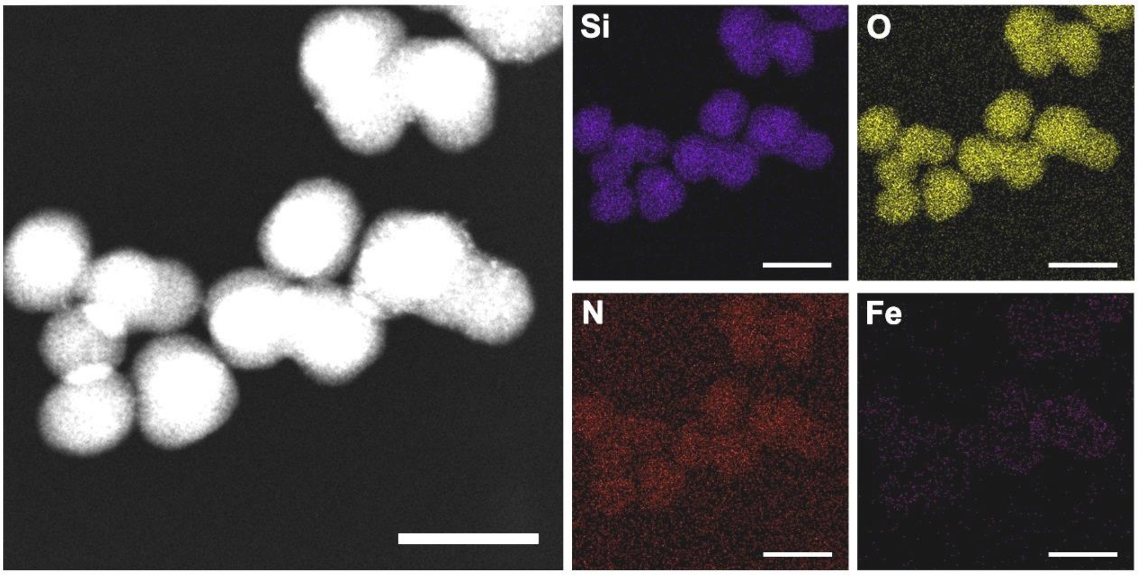

Figure. S10. HAADF-STEM image and the corresponding element mapping images of cSMNs.

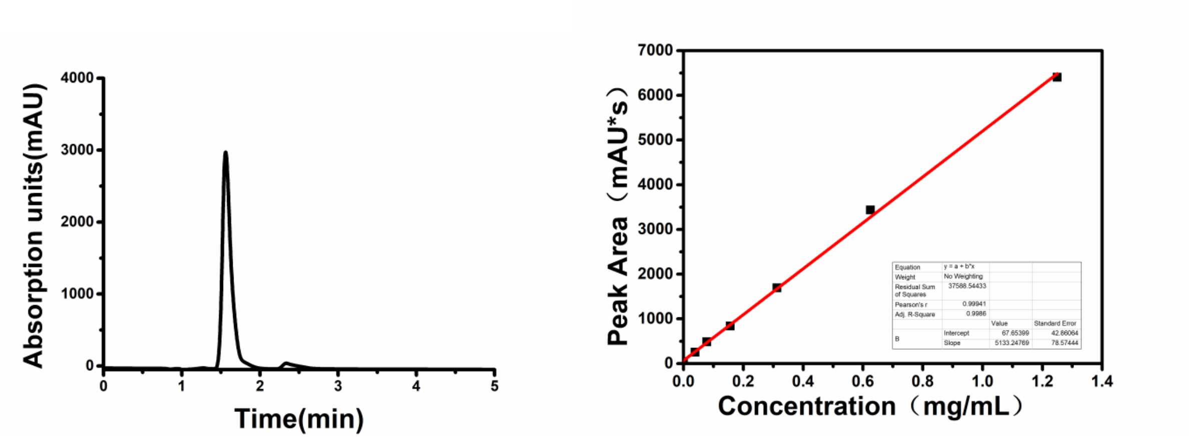

Figure. S11. Chromatogram of the captopril standard (0.2 mg/mL), and the peak area of various concentration of captopril from 0 mg/mL to 1.25 mg/mL at the retention time of 1.5 min.

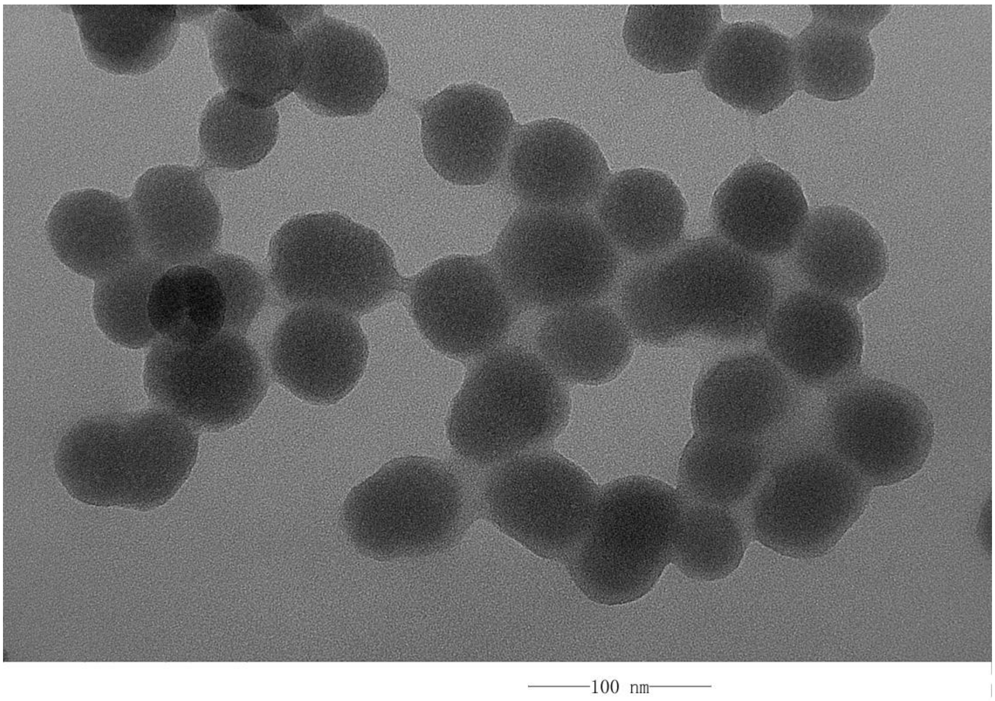

Figure. S12. The orignial TEM image of mD@cSMN nano-vaccines.

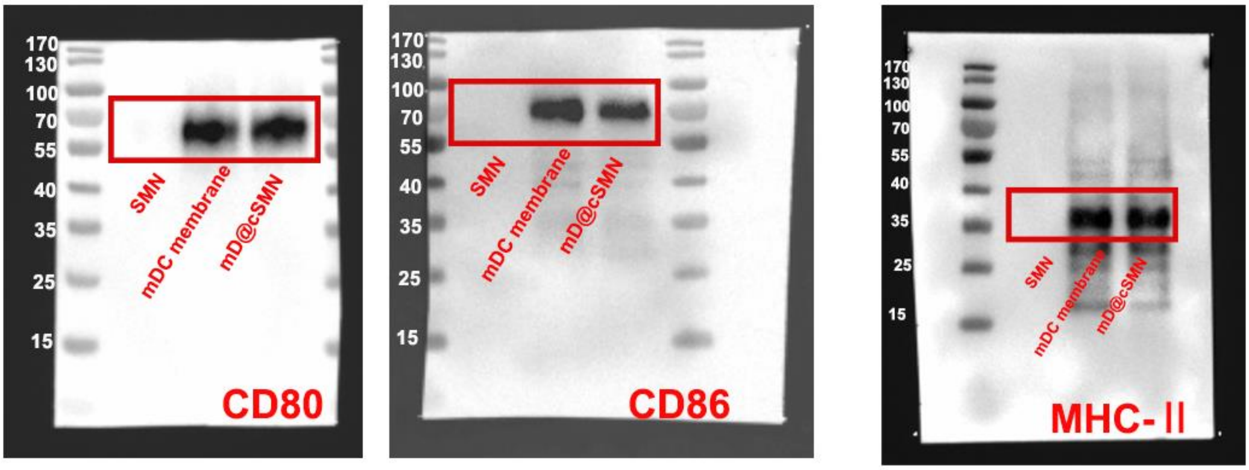

Figure. S13. Original data of Western blotting analysis of membrane-specific protein markers (CD80, CD86 and MHC-II).

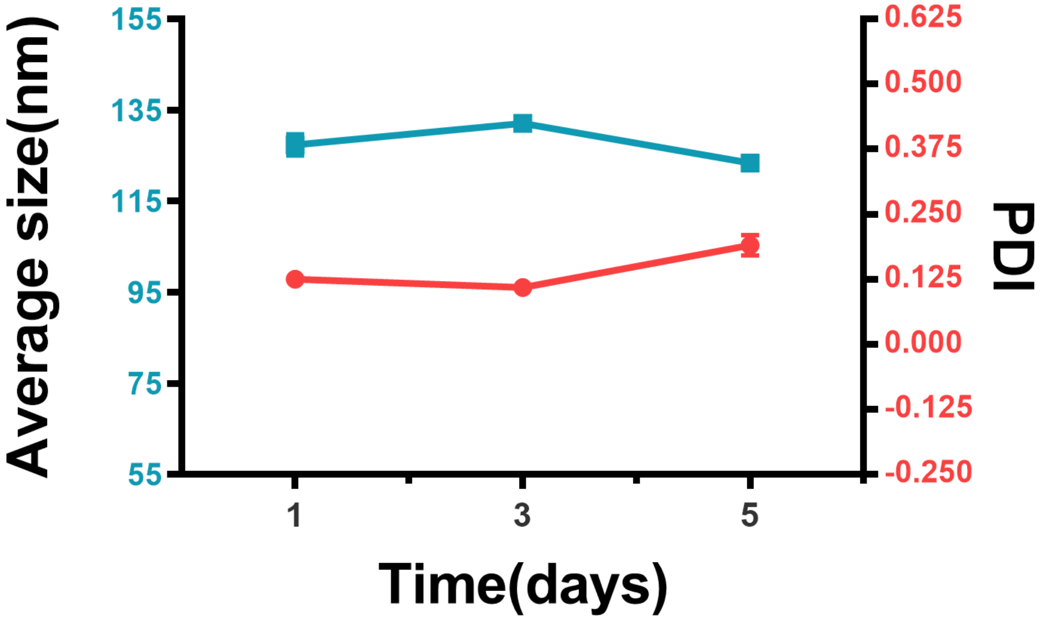

Figure. S14. Average size and PDI of mD@cSMN nano-vaccines after storage at room temperature for 1, 3, and 5 days, respectively. The data was obtained from DLS. Data are presented as mean ± SEM (n=3).

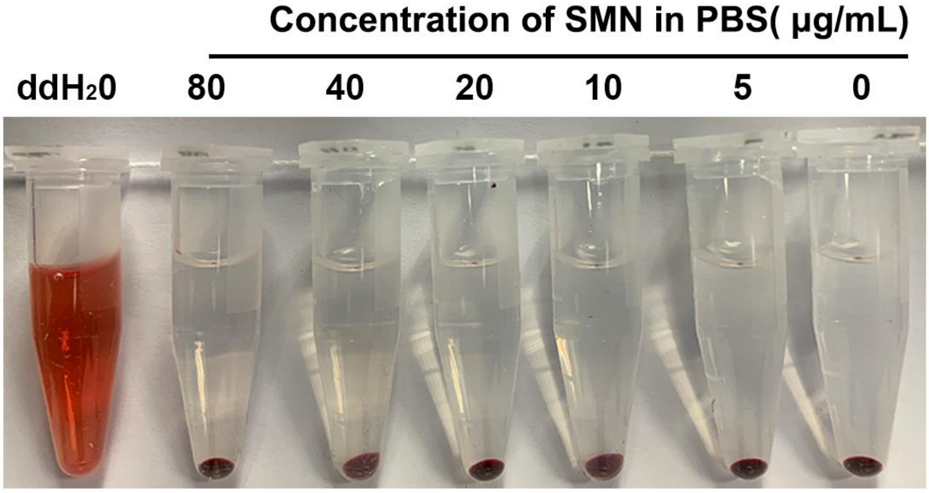

Figure. S15. Hemolysis assay of various concentrations of SMNs after co-incubation with mouse red blood cells, and then imaged by digital camera.

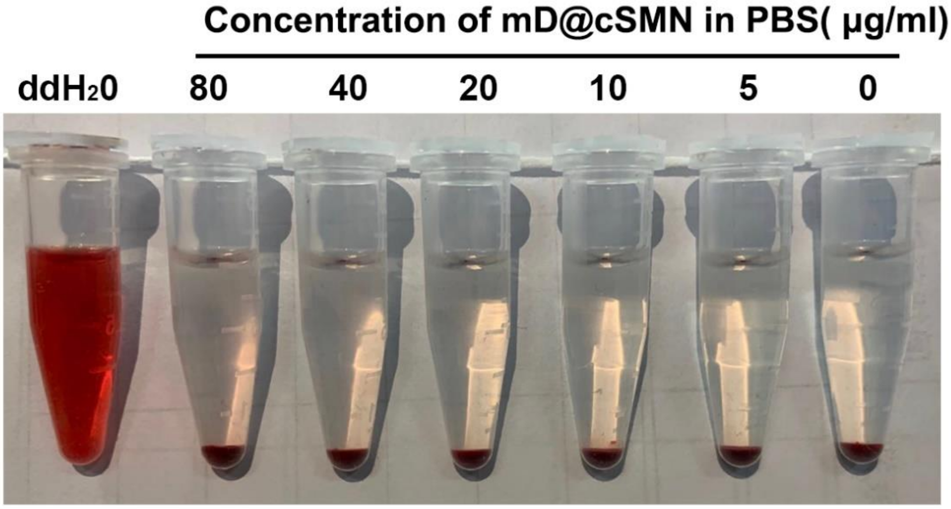

Figure. S16. Hemolysis assay of various concentrations of mD@cSMNs after co-incubation with mouse red blood cells, and imaged by digital camera.

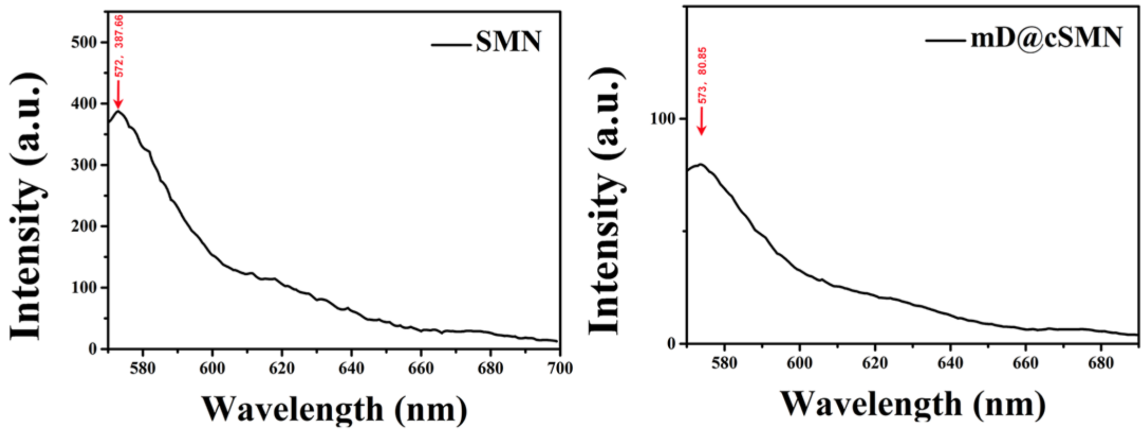

Figure. S17. The emisstion of Dylight550 labeled SMN or mD@cSMN.

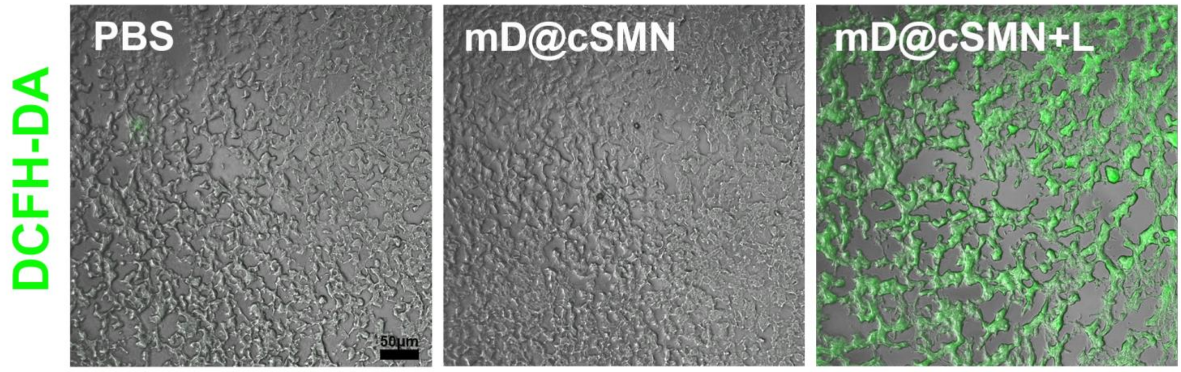

Figure. S18. The in vivo ROS generation ability of mD@cSMN with or without NIR irradiation (670 nm, 0.1 W/cm^2) for 5 min. The PBS treated tumor without NIR laser irradiation was as the control. The green fluorescence represented as DCFH-DA (ROS indicator), scale bar, 50μm.

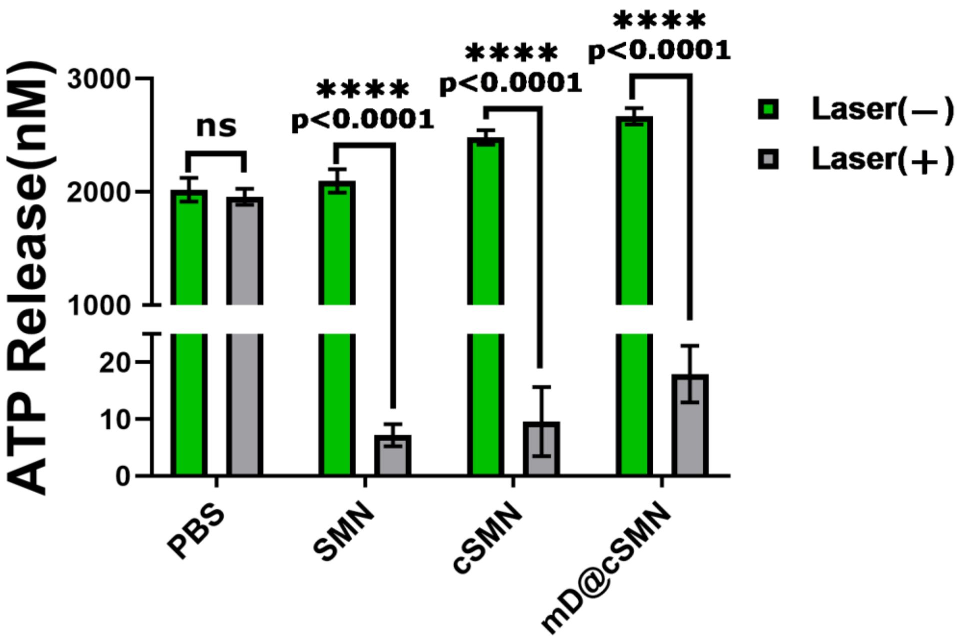

Figure. S19. The ATP release detection from PBS, SMN, cSMN, or mD@cSMN treated H22 cancer cells with or without laser irradiation, and then analyzed by ATP Assay Kit. The statistical analysis was performed with two-tail paired Student's *t-test* analysis, ns means $p > 0.05$, ****$p < 0.0001$. Data are presented as mean ± SEM (n =3).

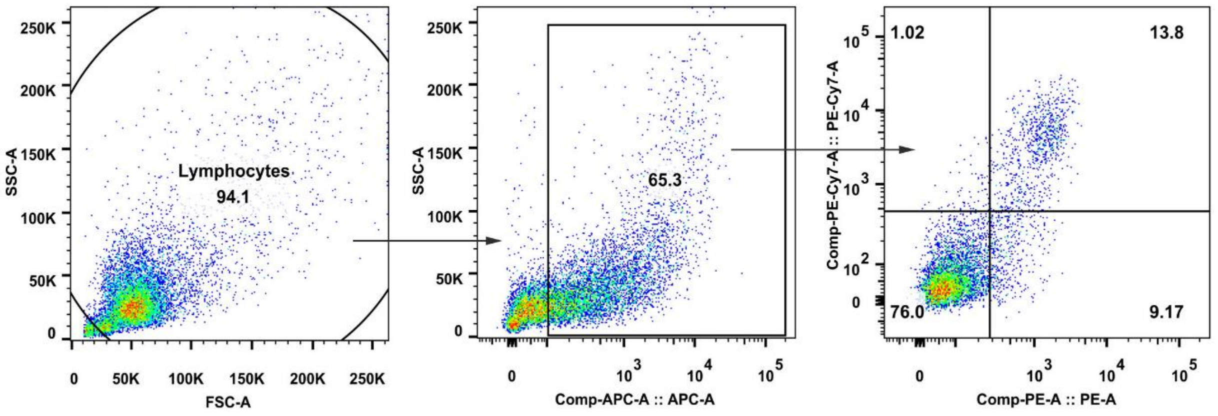

Figure. S20. Identify the maturation of BMDCs after receiving different treatment through FACS with staining anti-CD11c-APC, anti-CD80-PE, and anti-CD86-PE-Cy7 antibodies.

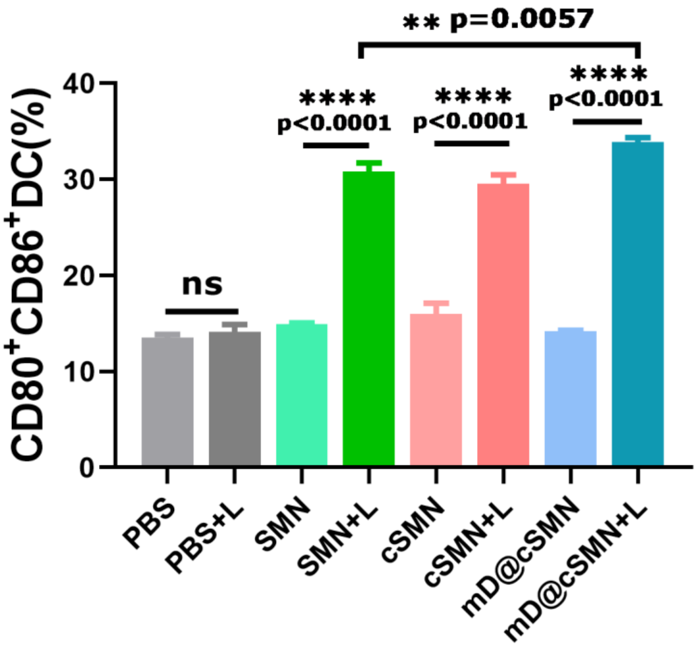

Figure. S21. The expression of CD80+ and CD86+ in BMDCs after receiving different treatments as indicated *in vitro*. The statistical analysis was performed with ANOVA analysis, ***p < 0.01, ****p < 0.0001*. Data are presented as mean ± SEM (n =3).

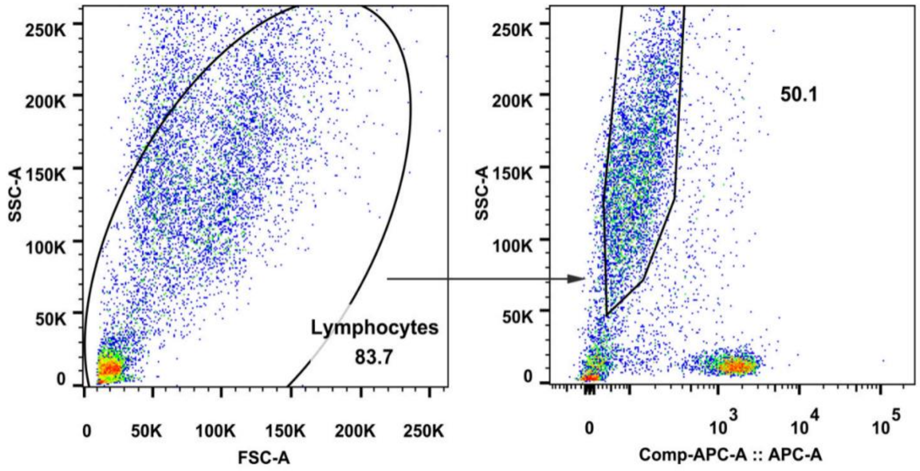

Figure. S22. The cytotoxicity of T cells to H22 cells after staining with anti-CD3-APC antibodies, Annexin V-APC and PI.

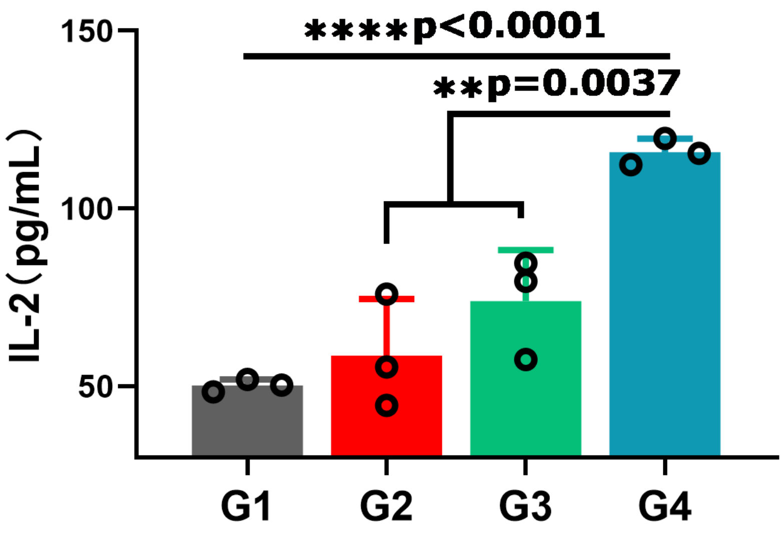

Figure. S23. The secretion of IL-2 from activated T cells was checked by ELISA kit after 72 h of post-incubation with H22 cells. The statistical analysis was performed with ANOVA analysis, $^{**}p < 0.01$, $^{****}p < 0.0001$. Data are presented as mean ± SEM (n =3).

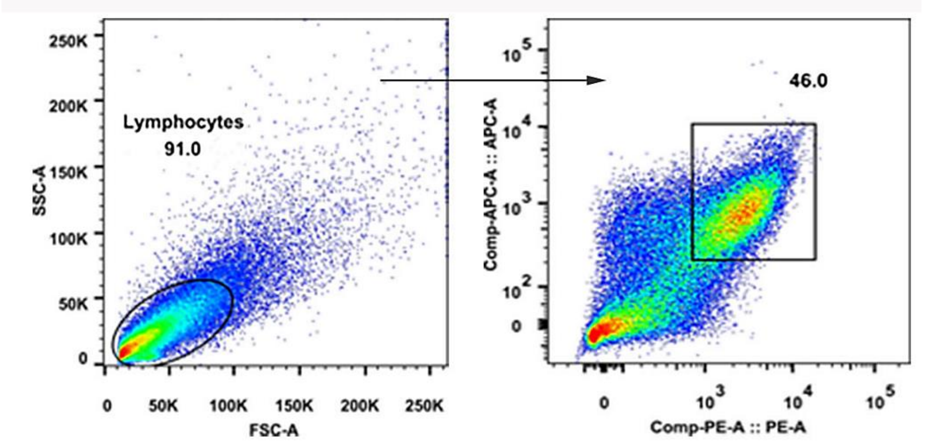

Figure. S24. Identify the state of neutrophiles in tumors after receiving different treatment through FACS with staining anti-CD11b-APC and anti-Ly-6G/Ly-6C-PE antibodies.

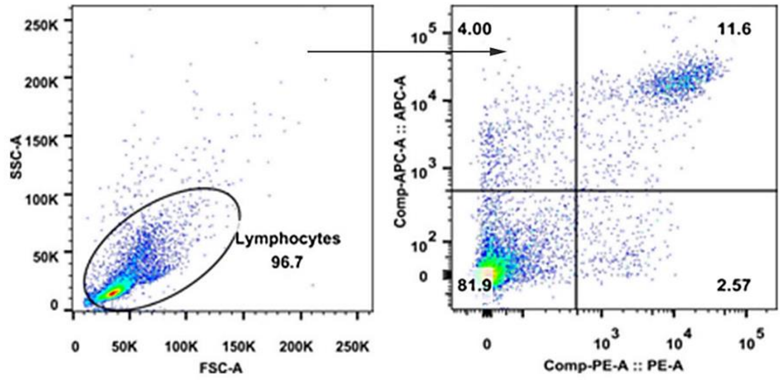

Figure. S25. Identify the state of neutrophiles in the spleen after receiving different treatment through FACS with staining anti-CD11b-APC and anti-Ly-6G/Ly-6C-PE antibodies.

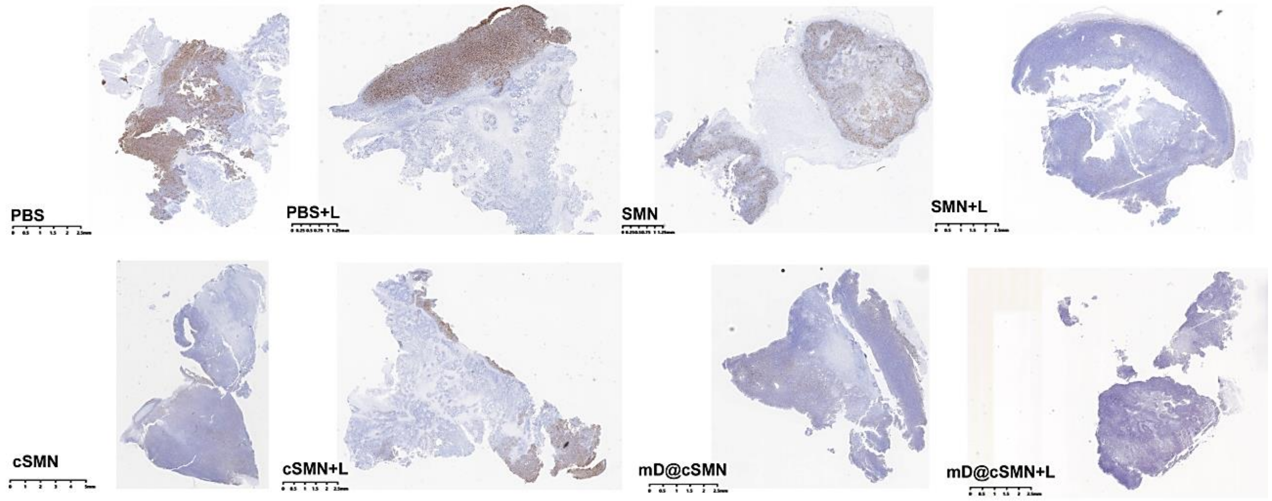

Figure. S26. The raw data of ki67 staining of tumor slice at the 20th d after receiving different treatment as indicated, 1.25, 2.5 or 5 mm.

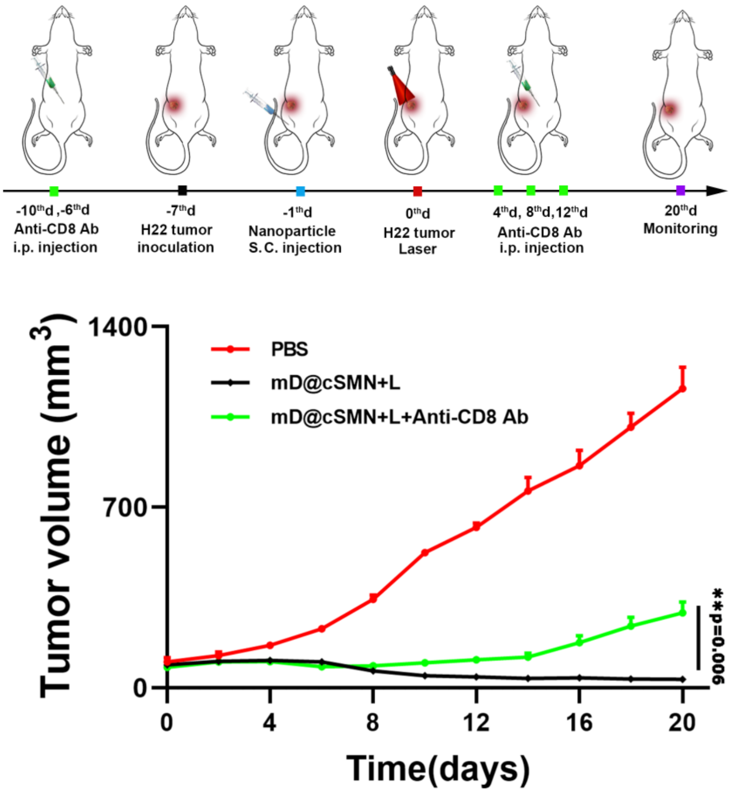

Figure. S27. Schematic illustration of the process of administration mD@cSMN, the laser irradiation and the T cell depletion by anti-CD8 antibody. The average tumor volume change of mice after receiving different treatment as indicated in H22 tumor bearing mice model. The statistical analysis was performed with ANOVA analysis, ***$p < 0.01$*. Data are presented as mean ± SEM (n =4).

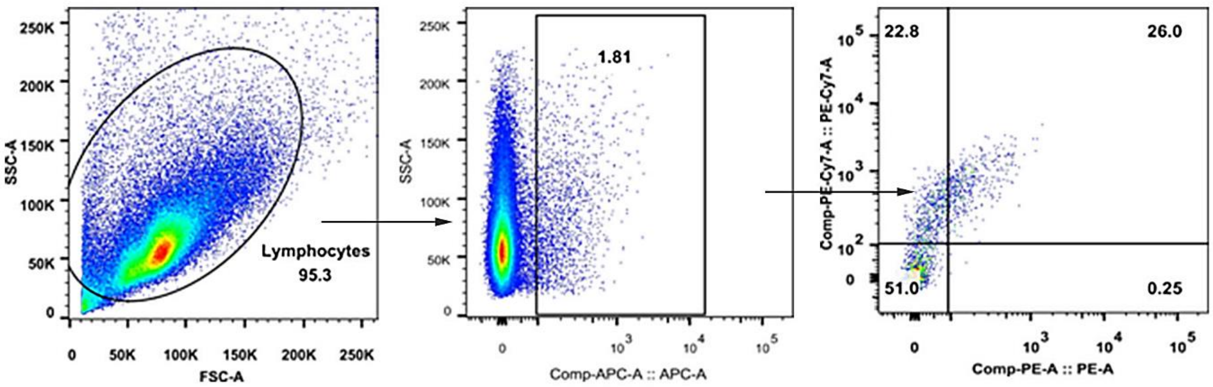

Figure. S28. Identify the maturation of DCs in LNs after receiving different treatment through FACS with staining anti-CD11c-APC, anti-CD80-PE, and anti-CD86-PE-Cy7 antibodies.

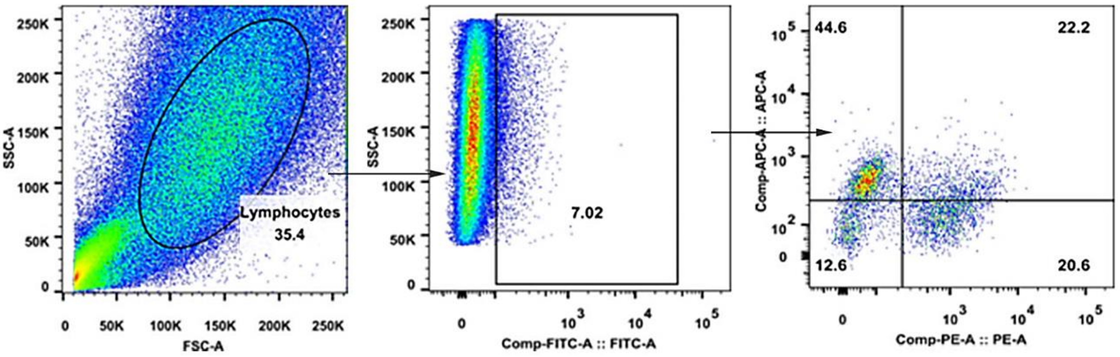

Figure. S29. Identify the CD8+T cells in tumors after receiving different treatment through FACS with staining anti-CD3-FITC, anti-CD8-PE, and anti-CD137-APC antibodies.

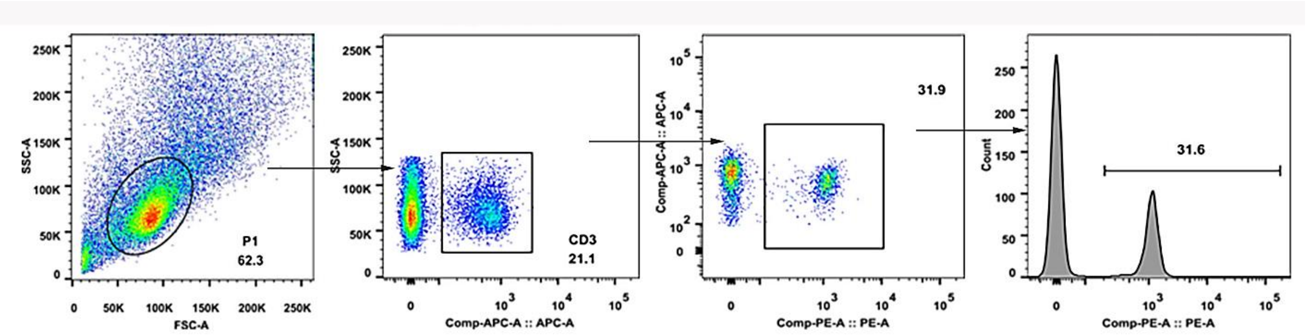

Figure. S30. Identify the CD8+T cells in the spleen after receiving different treatment through FACS with staining anti-CD3-APC and anti-CD8-PE antibodies.

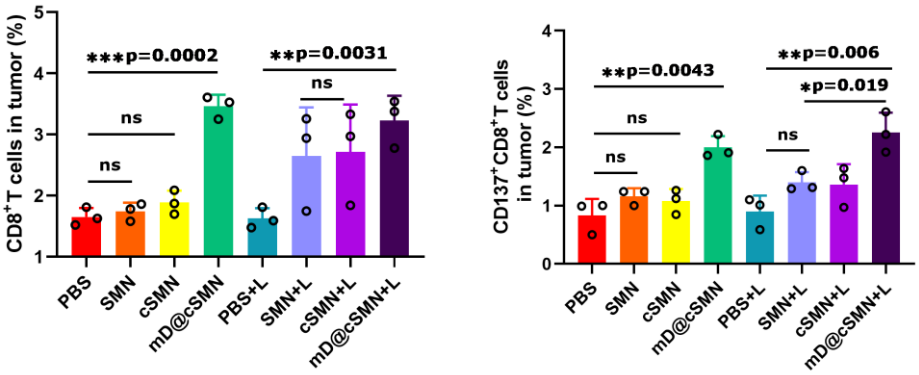

Figure. S31. The population of CD8+T cells and CD137+CD8+T cells in tumor after receiving different treatment as indicated. The statistical analysis was performed with ANOVA analysis, $^{**}p < 0.01$, $^{****}p < 0.0001$. Data are presented as mean ± SEM (n =3).

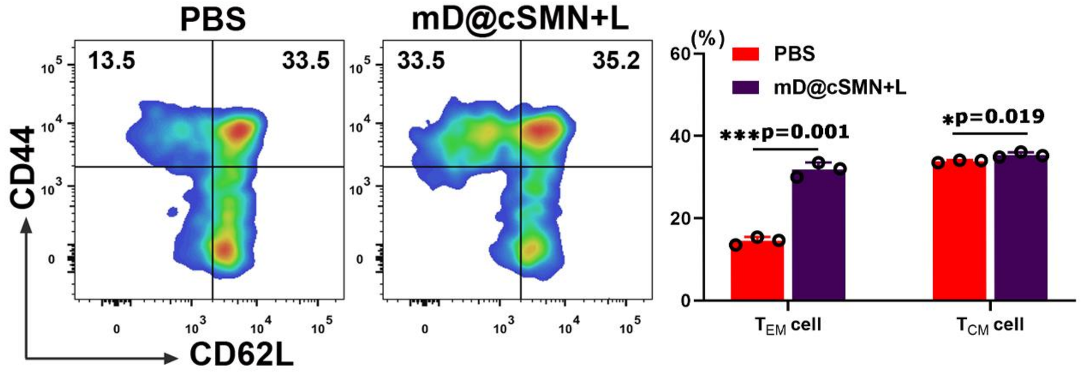

Figure. S32. FACS analysis of the T_{CM} and T_{EM} in tumor-draining LNs isolated from mice after mD@cSMN+NIR treatment by staining CD44 and CD62L. The statistical analysis was performed with two-tail paired Student's *t-test* analysis, $*p < 0.05$, $***p < 0.001$. Data are presented as mean ± SEM (n =3).

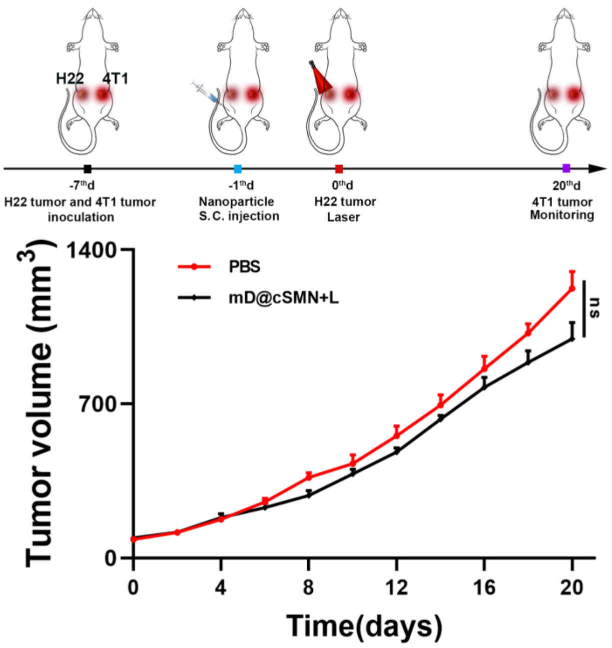

Figure S33. Schematic illustration of the process of administration mD@cSMN with NIR irradiation of H22 tumor (left), and 4T1 tumor without NIR laser irradiation (right) to evaluate the specific antitumor immune responses. The average 4T1 tumor volume change of mice after PBS or mD@cSMN treatment (right). Data are presented as mean ± SEM (n =4).

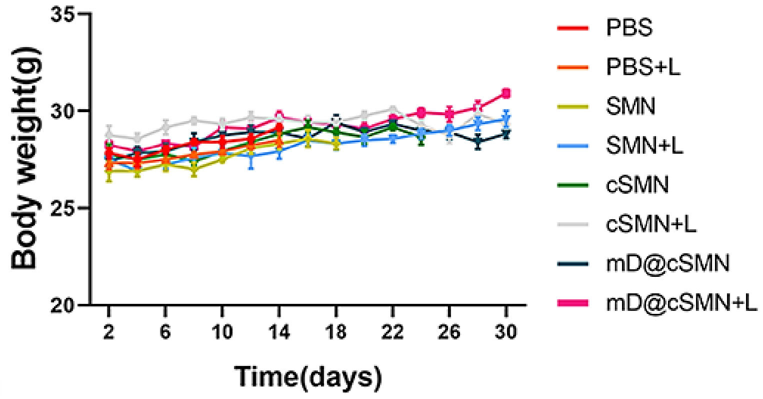

Figure 34. Body weight of mice during the different treatments as indicated. Data are presented as mean ± SEM (n=6).

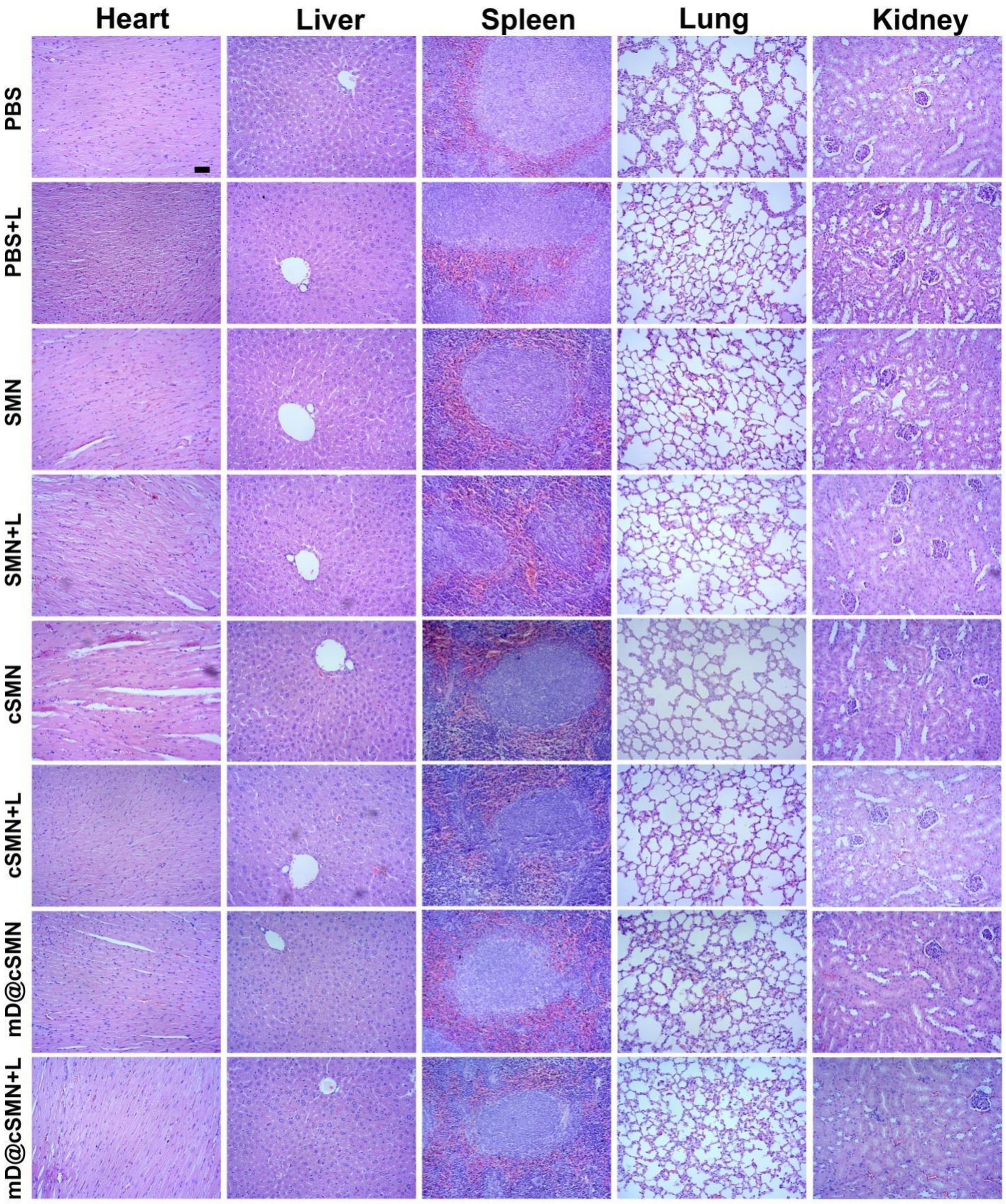

Figure. S35. H&E staining of mice during the different treatments as indicated at 20 days. Scale bar, 100 μm.
